# Synthesis of β-d-galactopyranoside-Presenting Glycoclusters, Investigation of Their Interactions with *Pseudomonas aeruginosa* Lectin A (PA-IL) and Evaluation of Their Anti-Adhesion Potential

**DOI:** 10.3390/biom9110686

**Published:** 2019-11-01

**Authors:** Lenka Malinovská, Son Thai Le, Mihály Herczeg, Michaela Vašková, Josef Houser, Eva Fujdiarová, Jan Komárek, Petr Hodek, Anikó Borbás, Michaela Wimmerová, Magdolna Csávás

**Affiliations:** 1 Central European Institute of Technology, Masaryk University, Kamenice 5, 625 00 Brno, Czech Republic; malinovska@mail.muni.cz (L.M.); houser@mail.muni.cz (J.H.); honzakomarek@mail.muni.cz (J.K.); 2 National Centre for Biomolecular Research, Faculty of Science, Masaryk University, Kotlářská 2, 611 37 Brno, Czech Republic; eva.fujdiarova@mail.muni.cz; 3 Department of Pharmaceutical Chemistry, University of Debrecen, Egyetem tér 1, H-4032 Debrecen, Hungary; le.thai.son@pharm.unideb.hu (S.T.L.); herczeg.mihaly@science.unideb.hu (M.H.); borbas.aniko@pharm.unideb.hu (A.B.); 4 Doctoral School of Pharmaceutical Sciences, University of Debrecen, Egyetem tér 1, H-4032 Debrecen, Hungary; 5 Research Group for Oligosaccharide Chemistry of Hungarian Academy of Sciences, Egyetem tér 1, H-4032 Debrecen, Hungary; 6 Department of Biochemistry, Faculty of Science, Charles University, Albertov 2030, 128 40 Prague 2, Czech Republic; michael.vaskova@gmail.com (M.V.); petr.hodek@natur.cuni.cz (P.H.); 7 Department of Biochemistry, Faculty of Science, Masaryk University, Kotlářská 2, 611 37 Brno, Czech Republic

**Keywords:** *Pseudomonas aeruginosa*, cystic fibrosis, lectin, d-galactosides, multivalency

## Abstract

*Pseudomonas aeruginosa* is an opportunistic human pathogen associated with cystic fibrosis. This bacterium produces, among other virulence factors, a soluble d-galactose-specific lectin PA-IL (LecA). PA-IL plays an important role in the adhesion to the host cells and is also cytotoxic. Therefore, this protein is an interesting therapeutic target, suitable for inhibition by carbohydrate-based compounds. In the current study, β-d-galactopyranoside-containing tri- and tetravalent glycoclusters were synthesized. Methyl gallate and pentaerythritol equipped with propargyl groups were chosen as multivalent scaffolds and the galactoclusters were built from the above-mentioned cores by coupling ethylene or tetraethylene glycol-bridges and peracetylated propargyl β-d-galactosides using 1,3-dipolar azide-alkyne cycloaddition. The interaction between galactoside derivatives and PA-IL was investigated by several biophysical methods, including hemagglutination inhibition assay, isothermal titration calorimetry, analytical ultracentrifugation, and surface plasmon resonance. Their ability to inhibit the adhesion of *P. aeruginosa* to bronchial cells was determined by ex vivo assay. The newly synthesized multivalent galactoclusters proved to be significantly better ligands than simple d-galactose for lectin PA-IL and as a result, two representatives of the dendrimers were able to decrease adhesion of *P. aeruginosa* to bronchial cells to approximately 32% and 42%, respectively. The results may provide an opportunity to develop anti-adhesion therapy for the treatment of *P. aeruginosa* infection.

## 1. Introduction

Lectins are specific carbohydrate-binding proteins of a non-immune origin. Lectins from the pathogens could be important virulence factors involved in recognition and adhesion processes between pathogens and hosts [1]. Therefore, lectins are promising therapeutic targets which could be inhibited by carbohydrate-based inhibitors [2]. Lectins are usually multivalent proteins, containing several binding sites per molecule and/or forming oligomers. They frequently display an avidity effect, resulting in significantly increased affinity towards glycosylated surfaces. Consequently, the multivalent compounds with multiple carbohydrate moieties available are considered to be a possible method to efficiently inhibit lectins [3].

The Gram-negative bacterium *Pseudomonas aeruginosa* is an important opportunistic pathogen causing lung infections in immunocompromised individuals, especially cystic fibrosis patients. It is the most common pathogen associated with this hereditary disease [4]. *P. aeruginosa* produces soluble d-galactose-specific lectin PA-IL (LecA) [5,6]. PA-IL is a tetrameric calcium-dependent lectin with one binding site per monomer. Each binding site contains one calcium ion directly involved in the carbohydrate recognition and binding [7]. PA-IL is considered to be a significant virulence factor [8]. In addition to its supposed primary role as an adhesin, the lectin is involved in the biofilm formation [9] and is cytotoxic. Specifically, PA-IL displayed toxicity to respiratory epithelial cells in primary culture [10]. Due to its importance, several types of multivalent inhibitors were designed and tested against PA-IL, including fullerene-based glycoclusters [11], glycodendrimers [12], glycopeptide dendrimers [13], resorcin[4]arene-based glycoclusters [14], inhibitors based on porphyrin scaffold and β-peptoids [15], glycosylated poly(phenylacetylene)s [16], glycoconjugates based on a calix[4]arene scaffold [17,18] and on a pentaerythritol core [19]. Furthermore, the d-galactose surface-modified polymeric nanoparticles [20] and glycodendrimer micelles [21] were prepared.

In many cases, the efficiency of multivalent carbohydrate-based inhibitors is based on the chelate effect—the intramolecular binding of multivalent ligand to several binding sites of lectin. However, the multivalent inhibitors can also cross-link lectin molecules by intermolecular binding, possibly resulting in the formation of large aggregates and subsequent precipitation of the complexes [22]. Considering PA-IL, it was suggested that tetravalent inhibitors capable of lectin cross-linking are the most suitable for the inhibition of *Pseudomonas aeruginosa* biofilm formation [23]. The ability of multivalent compounds to cross-link lectins can also lead to cross-linking and aggregation of bacteria cells presenting these lectins on their surfaces [18,24,25]. This aggregation can serve as proof that inhibitors are able to interact with native lectins on the cells surfaces; however, it can also interfere with biofilm formation. A negative correlation to biofilm formation was observed for the aggregation of *Pseudomonas aeruginosa* cells [26].

In the current paper, we describe the efficient synthesis of β-d-galactopyranoside-presenting oligovalent glycoclusters and their testing as inhibitors of the lectin PA-IL. Their inhibitory potency was determined by hemagglutination inhibition assay and surface plasmon resonance. Interactions were characterized using isothermal titration calorimetry and the cross-linking ability was examined by analytical ultracentrifugation and experiments with whole *Pseudomonas aeruginosa* cells. Selected compounds were further tested for their ability to inhibit the adhesion of *P. aeruginosa* to epithelial bronchial cells derived from a cystic fibrosis patient in an ex vivo assay.

## 2. Materials and Methods

### 2.1. General Methods

Optical rotations were measured at room temperature with a Perkin-Elmer 241 automatic polarimeter (PerkinElmer, Waltham, MA, USA). TLC analysis was performed on Kieselgel 60 F_254_ silica gel plates (Merck, Kenilworth, NJ, USA) with visualization by immersing in a sulfuric-acid solution (5% in EtOH, VWR International Ltd., Radnor, PA, USA) followed by heating. Column chromatography was performed on silica gel 60 (Merck 0.063–0.200 mm) and flash column chromatography was performed on silica gel 60 (Merck 0.040–0.063 mm). Gel filtration was performed on Sephadex G-25 using methanol as the eluent. Organic solutions were dried over MgSO_4_ and concentrated under vacuum. The ^1^H (400 and 500 MHz) and ^13^C NMR (100.28, 125.76 MHz) spectra were recorded with Bruker DRX-400 and Bruker Avance II 500 spectrometers (Billerica, MA, USA). Chemical shifts are referenced to Me_4_Si or DSS (0.00 ppm for ^1^H) and to solvent signals (CDCl_3_: 77.00 ppm, CD_3_OD: 49.15 ppm for ^13^C). MS (MALDI-TOF) analysis was carried out in positive reflectron mode with a BIFLEX III mass spectrometer (Billerica, MA, USA) with delayed-ion extraction. The matrix solution was a saturated solution of 2,4,6-trihydroxy-acetophenone (THAP) in CH_3_CN.

### 2.2. Synthesis

#### 2.2.1. General Method A for Azide-Alkyne Click Reaction

Et_3_N (1 equiv./alkyne) and Cu(I)I (0.1 equiv./alkyne) were added to a stirred solution of alkyne (1.5 equiv/azide moiety) and azide in CH_3_CN under an argon atmosphere and stirred overnight at room temperature. The reaction mixture was evaporated and the crude product was purified by flash column chromatography to give the desired compound.

#### 2.2.2. General Method B for Zemplén-Deacetylation

A catalytic amount of NaOMe (pH = 9) was added to a stirred solution of ester (0.2 mmol) in dry MeOH (5 mL) and stirred overnight at room temperature. The reaction mixture was neutralized with Amberlite IR-120 H^+^ ion-exchange resin, filtered and evaporated, then the crude product was purified by flash column chromatography and gel filtration to give the desired compound.

#### 2.2.3. Compound **3**

Azide compound **1** (100 mg, 0.10 mmol) and alkyne **2** (169 mg, 0.44 mmol) were reacted in CH_3_CN according to the general method A. The crude product was purified by flash column chromatography (95:5 CH_2_Cl_2_:MeOH) to give compound **3** (118 mg, 55%) as a colourless syrup. [α]^24^_D_ –21.96 (*c* 0.46, CHCl_3_); R*_f_* 0.33 (95:5 CH_2_Cl_2_: MeOH); ^1^H NMR (400 MHz, CDCl_3_): *δ* = 7.94, 7.90, 7.72 (3 x s, 6H, 6 x CH triazole), 7.44 (s, 2H, arom), 5.40 (d, *J* = 2.9 Hz, 3H, skeleton proton), 5.23 (s, 6H, 3 x C*H*_2_), 5.20–5.18 (m, 3H, 3 x skeleton protons), 5.02 (dd, *J* = 3.4 Hz, *J* = 10.4 Hz, 3H, 3 x skeleton protons), 4.94 (d, *J* = 12.5 Hz, 3H, 3 x H-6a), 4.80 (d, *J* = 12.5 Hz, 3H, 3 x H-6b), 4.68 (d, *J* = 7.9 Hz, 3H, 3 x H-1), 4.59–4.50 (m, 11H, 3 x H-5, 4 x C*H*_2_), 4.18–4.15 (m, 6H, 3 x C*H*_2_), 3.99–3.84 (m, 19H, 8 x C*H*_2_, COOC*H*_3_), 3.61-3.55 (m, 24H, 12 x C*H*_2_), 2.15, 2.06, 1.97 (3 x s, 36H, 12 x C*H*_3_ acetyl) ppm; ^13^C NMR (100 MHz, CDCl_3_) *δ* = 170.4, 170.2, 170.1, 169.5 (12C, 12 x *C*O acetyl), 166.3 (1C, *C*OOCH_3_), 152.0 (2C, 2 x Cq arom), 144.0, 148.8, 143.1, 141.6 (6C, 6 x Cq triazole), 125.7 (2C, 2 x Cq arom), 124.9, 124.5, 124.0 (6C, 6 x *C*H triazole), 109.2 (2C, arom), 100.3, 100.2 (3C, 3 x C-1), 70.9, 70.8, 68.8, 67.1 (12C, 3 x skeleton carbons), 70.5, 70.4, 69.3 (18C, 18 x *C*H_2_ TEG), 63.0, 62.8, 62.7 (3C, 3 x C-6), 61.3 (6C, 6 x *C*H_2_ propargyl), 52.4 (1C, COO*C*H_3_), 50.3, 50.2, 50.1 (6C, 6 x N*C*H_2_ TEG), 20.7, 20.6 (12C, 12 x *C*H_3_ acetyl) ppm; MALDI-TOF (positive ion): *m/z* calcd for C_92_H_128_N_18_NaO_44_: 2211.82 [M + Na]^+^ Found: 2211.78.

#### 2.2.4. Compound **4**

Compound **3** (86 mg, 0.04 mmol) was deacetylated according to general method B. The crude product was purified by flash column chromatography (7:3 CH_3_CN:H_2_O) to give compound **4** (48 mg, 73%) as a colourless syrup. [α]^24^_D_—6.4 (*c* 0.14, MeOH); R*_f_* 0.34 (7:3 CH_3_CN:H_2_O); ^1^H NMR (400 MHz, D_2_O): *δ* = 8.00, 7.92, 7.73 (3 x s, 6H, 6 x C*H* triazole), 7.29 (s, 2H, arom), 5.08 (s, 4H, 2 x C*H*_2_ propargyl), 5.01 (s, 2H, C*H*_2_ propargyl), 4.84-4.65 (m, 6H, 3 x C*H*_2_ propargyl), 4.51-4.38 (m, 12H, 6 x NC*H*_2_ TEG), 4.34 (d, *J* = 7.7 Hz, 3H, 3 x H-1), 3.81–3.70 (m, 22H, 3 x H-4, COOC*H*_3_, 8 x OC*H*_2_ TEG), 3.69-3.61 (m, 6H, 3 x H-6a,b), 3.56 (dd, *J* = 4.4 Hz, *J* = 7.5 Hz, 3H, 3 x H-5), 3.50 (dd, *J* = 3.4 Hz, *J* = 9.9 Hz, 3H, 3 x H-3), 3.44–3.32 (m, 23H, 3 x H-2, 10 x OC*H*_2_ TEG) ppm; ^13^C NMR (100 MHz, D_2_O) *δ* = 168.7 (1C, *C*OOCH_3_), 152.4 (2C, 2 x Cq arom), 144.6, 143.8, 141.3 (6C, 6 x Cq triazole), 126.7, 126.4 (6C, 6 x *C*H triazole), 126.5 (2C, 2 x Cq arom), 110.1 (2C, arom), 103.0 (3C, 3 x C-1), 76.2 (3C, 3 x C-5), 73.8 (3C, 3 x C-3), 71.6 (3C, 3 x C-2), 70.7, 70.6, 70.4, 70.0, 69.6 (18C, 18 x *C*H_2_ TEG), 69.5 (3C, 3 x C-4), 66.0, 62.9 61.7 (6C, 6 x *C*H_2_ propargyl), 61.9 (3C, 3 x C-6), 53.9 (1C, COO*C*H_3_), 51.1, 51.0, 50.9 (6C, 6 x N*C*H_2_ TEG) ppm; MALDI-TOF (positive ion): *m/z* calcd for C_68_H_104_N_18_NaO_32_: 1707.70 [M + Na]^+^ Found: 1707.65.

#### 2.2.5. Compound **6**

Azide compound **5** (85 mg, 0.05 mmol) and alkyne **2** (111 mg, 0.29 mmol) were reacted in CH_3_CN according to general method A. The crude product was purified by flash column chromatography (95:5 CH_2_Cl_2_:MeOH) to give compound **6** (79 mg, 42%) as a colourless syrup. [α]^24^_D_—16.9 (*c* 0.13, CHCl_3_); R*_f_* 0.38 (95:5 CH_2_Cl_2_: MeOH); ^1^H NMR (400 MHz, CDCl_3_) *δ* = 7.73, 7.72 (2 x s, 8H, 8 x C*H* triazole), 5.40 (d, *J* = 2.8 Hz, 4H, 4 x H-4), 5.20 (dd, *J* = 8.0 Hz, *J* = 10.4 Hz, 4H, 4 x H-2), 5.02 (dd, *J* = 3.4 Hz, *J* = 10.4 Hz, 4H, 4 x H-3), 4.95 (d, *J* = 12.5 Hz, 4H, 4 x C*H*_2_a), 4.80 (d, *J* = 12.5 Hz, 4H, 4 x C*H*_2_b), 4.68 (d, *J* = 8.0 Hz, 4H, 4 x H-1), 4.58–4.53 (m, 20H, 8 x C*H*_2_ propargyl, 2 x C*H*_2_ TEG), 4.19–4.15 (m, 8H, 4 x H-6a,b), 3.99-3.94 (m, 4H, 4 x H-5), 3.90-3.86 (m, 20H, 10 x C*H*_2_ TEG), 3.62-3.58 (m, 32H, 16 x C*H*_2_ TEG), 3.46 (s, 8H, 4 x C*H*_2_ pentaerythritol), 2.15, 2.06, 1.98, 1.97 (4 x s, 48H, 16 x C*H*_3_ acetyl) ppm; ^13^C NMR (100 MHz, CDCl_3_) *δ* = 170.4, 170.2, 170.1, 169.5 (16C, 16 x *C*O acetyl), 145.0, 143.9 (8C, Cq triazole), 124.0, 123.7 (8C, *C*H triazole), 100.3 (4C, 4 x C-1), 70.9, 70.8, 68.8, 67.1 (16C, 4 x skeleton carbons), 70.5, 70.4, 69.4, 69.3, 69.1 (28C, 24 x O*C*H_2_ TEG, 4 x *C*H_2_ pentaerythritol), 64.9 (4C, 4 x *C*H_2_ propargyl), 62.8 (4C, 4 x C-6), 61.3 (4C, 4 x *C*H_2_ propargyl), 50.3, 50.1 (8C, 8 x N*C*H_2_ TEG), 45.3 (1C, Cq pentaerythritol), 20.7, 20.6, 20.5 (16C, 16 x *C*H_3_ acetyl) ppm;MALDI-TOF (positive ion): *m/z* calcd for C_117_H_172_N_24_NaO_56_: 2832.12 [M + Na]^+^ Found: 2832.15.

#### 2.2.6. Compound **7**

Compound **6** (78 mg, 0.28 mmol) was deacetylated according to general method B. The crude product was purified by flash column chromatography (7:3 CH_3_CN:H_2_O) to give compound **7** (44 mg, 73%) as a colourless syrup. [α]^24^_D_—2.5 (*c* 0.12, MeOH); R*_f_* 0.15 (7:3 CH_3_CN:H_2_O); ^1^H NMR (400 MHz, D_2_O) *δ* = 7.95, 7.89 (2 x s, 8H, 8 x C*H* triazole), 4.85-4.66 (m, 16H, 8 x C*H*_2_ propargyl), 4.45–4.44 (m, 16H, 4 x NC*H*_2_ TEG), 4.32 (d, *J* = 7.6 Hz, 4H, 4 x H-1), 3.80-3.77 (m, 20H, 4 x C-4, 8 x C*H*_2_ TEG), 3.68-3.59 (m, 8H, 4 x H-6a,b), 3.55-3.53 (m, 4H, 4 x H-5), 3.47 (dd, *J* = 3.3 Hz, *J* = 9.9 Hz, 4H, 4 x H-3), 3.43-3.37 (m, 36H, 4 x C-2, 16 x C*H*_2_ TEG), 3.30-3.27 (m, 8H, 4 x C*H*_2_ pentaerythritol) ppm; ^13^C NMR (100 MHz, D_2_O) *δ* = 125.7 (8C, *C*H triazole), 103.1 (4C, 4 x C-1), 76.2 (4C, 4 x C-5), 73.9 (4C, 4 x C-3), 71.7 (4C, 4 x C-2), 70.7, 70.6, 70.5, 69.7 (24C, 24 x O*C*H_2_ TEG), 69.6 (4C, 4 x C-4), 69.3 (4C, 4 x *C*H_2_ pentaerythritol), 62.7 (8C, 8 x *C*H_2_ propargyl), 62.0 (4C, 4 x C-6), 51.0 (8C, 8 x N*C*H_2_ TEG), 45.8 (1C, Cq pentaerythritol) ppm; MALDI-TOF (positive ion): *m/z* calcd for C_85_H_140_N_24_NaO_40_: 2159.96 [M + Na]^+^ Found: 2159.95.

#### 2.2.7. Compound **9**

Azide compound **8** (50 mg, 0.08 mmol) and alkyne **2** (139 mg, 0.36 mmol) were reacted in CH_3_CN according to general method A. The crude product was purified by flash column chromatography (97:3 CH_2_Cl_2_:MeOH) to give compound **9** (125 mg, 89%) as a colourless syrup. [α]^24^_D_—22.9 (*c* 0.17, CHCl_3_); R*_f_* 0.11 (97:3 CH_2_Cl_2_: MeOH); ^1^H NMR (400 MHz, CDCl_3_) *δ* = 7.84, 7.76, 7.51, 7.50 (4 x s, 6H, 6 x C*H* triazole), 7.40 (s, 2H, arom), 5.39 (s, 3H, 3 x skeleton protons), 5.20–4.94 (m, 24H, 6 x C*H*_2_ propargyl, 6 x C*H*_2_ ethylene glycol), 4.91-4.88 (m, 3H, 3 x skeleton protons), 4.76-4.71 (m, 3H, 3 x skeleton protons), 4.63–4.60 (m, 3H, 3 x H-1), 4.19-4.09 (m, 6H, 3 x H-6a,b), 3.98-3.97 (m, 3H, 3 x H-5), 3.90 (s, 3H, COOC*H*_3_), 2.14, 2.04, 1.99, 1.97 (4 x s, 36H, 12 x C*H*_3_ acetyl) ppm; ^13^C NMR (100 MHz, CDCl_3_) *δ* = 170.5, 170.3, 170.1, 170.0, 169.6 (12C, 12 x CO acetyl), 166.2 (1C, *C*OOCH_3_), 151.8 (2C, 2 x Cq arom), 143.7, 141.4 (6C, 6 x Cq triazole), 124.2 (2C, 2 x Cq arom), 124.1 (6C, 6 x *C*H triazole), 109.1 (2C, arom), 100.4 (3C, 3 x C-1), 70.9, 70.8, 68.8, 67.1 (12C, 3 x skeleton carbons), 62.8, 62.7 (3C, 3 x C-6), 61.3 (6C, 6 x *C*H_2_ propargyl), 52.5 (1C, COO*C*H_3_), 49.6, 49.5 (6C, 6 x N*C*H_2_ ethylene glycol), 20.8, 20.7, 20.6 (12C, 12 x *C*H_3_ acetyl) ppm; MALDI-TOF (positive ion): *m/z* calcd for C_74_H_92_N_18_NaO_35_: 1815.59 [M + Na]^+^ Found: 1815.46.

#### 2.2.8. Compound **10**

Compound **9** (62 mg, 0.03 mmol) was deacetylated according to general method B. The crude product was purified by flash column chromatography (7:3 CH_3_CN:H_2_O) to give compound **10** (42 mg, 94%) as a colourless syrup. [α]^24^_D_—8.0 (*c* 0.10, H_2_O); R*_f_* 0.34 (7:3 CH_3_CN:H_2_O); ^1^H NMR (400 MHz, D_2_O) *δ* = 7.97, 7.90, 7.83 (3 x s, 6H, 6 x C*H* triazole), 7.32 (s, 2H, arom), 5.13–4.73 (m, 24H, 6 x C*H*_2_ propargyl, 6 x C*H*_2_ ethylene glycol), 4.39 (d, *J* = 7.2 Hz, 3H, 3 x H-1), 3.91–3.90 (m, 6H, 3 x H-4, COOC*H*_3_), 3.80-3.75 (m, 6H, 3 x H-6a,b), 3.56-3.60 (m, 6H, 3 x H-5, 3 x H-3), 3.54–3.50 (m, 3H, 3 x H-2) ppm; ^13^C NMR (100 MHz, D_2_O) *δ* = 168.8 (1C, *C*OOCH_3_), 152.1 (2C, 2 x Cq arom), 141.0 (6C, 6 x Cq triazole), 126.6 (2C, 2 x Cq arom), 125.5 (6C, 6 x *C*H triazole), 110.1 (2C, arom), 102.8 (3C, 3 x C-1), 76.2 (3C, 3 x C-5), 73.7 (3C, 3 x C-3), 71.6 (3C, 3 x C-2), 69.5 (3C, 3 x C-4), 62.5 (6C, 6 x *C*H_2_ propargyl), 61.9 (3C, 3 x C-6), 53.8 (1C, COO*C*H_3_), 50.9, 50.7 (6C, 6 x N*C*H_2_ ethylene glycol) ppm; MALDI-TOF (positive ion): *m/z* calcd for C_50_H_68_N_18_NaO_23_: 1311.46 [M + Na]^+^ Found: 1311.45.

#### 2.2.9. Compound **12**

Azide compound **11** (96 mg, 0.14 mmol) and alkyne **2** (315 mg, 0.82 mmol) were reacted in CH_3_CN according to general method A. The crude product was purified by flash column chromatography (95:5 CH_2_Cl_2_:MeOH) to give compound **12** (150 mg, 51%) as a colourless syrup. [α]^24^_D_—16.1 (*c* 0.23, CHCl_3_); R*_f_* 0.27 (95:5 CH_2_Cl_2_: MeOH); ^1^H NMR (400 MHz, CDCl_3_) *δ* = 7.59, 7.52 (2 x s, 8H, 8 x C*H* triazole), 5.41 (d, *J* = 2.1 Hz, 4H, 4 x H-4), 5.18 (dd, *J* = 8.1 Hz, *J* = 10.2 Hz, 4H, 4 x H-2), 5.04 (dd, *J* = 3.2 Hz, *J* = 10.4 Hz, 4H, 4 x H-3), 4.95-4.73 (m, 24H, 12 x C*H*_2_), 4.65 (d, *J* = 7.9 Hz, 4H, 4 x H-1), 4.51 (s, 8H, 4 x C*H*_2_), 4.21-4.11 (m, 8H, 4 x H-6a,b), 4.01-3.97 (m, 4H, 4 x H-5), 3.37 (s, 8H, 4 x C*H*_2_ pentaerythritol), 2.14, 2.05, 1.99, 1.98 (4 x s, 48H, 16 x C*H*_3_ acetyl) ppm; ^13^C NMR (100 MHz, CDCl_3_) *δ* = 170.4, 170.2, 170.0, 169.6 (16C, 16 x CO acetyl), 145.4, 144.2 (8C, 8 x Cq triazole), 124.0, 123.7 (8C, 8 x *C*H triazole), 100.3 (4C, 4 x C-1), 70.8, 70.7, 68.7, 67.1 (16C, 4 x skeleton carbons), 68.9 (4C, 4 x *C*H_2_ pentaerythritol), 64.6 (4C, 4 x *C*H_2_ propargyl), 62.6 (4C, 4 x C-6), 61.2 (4C, 4 x *C*H_2_ propargyl), 49.5, 49.3 (8C, 8 x N*C*H_2_ ethylene glycol), 45.1 (1C, Cq pentaerythritol), 20.8, 20.7, 20.6, 20.5 (16C, 16 x *C*H_3_ acetyl) ppm;MALDI-TOF (positive ion): *m/z* calcd for C_93_H_124_N_24_NaO_44_: 2303.81 [M + Na]^+^ Found: 2304.49.

#### 2.2.10. Compound **13**

Compound **12** (148 mg, 0.06 mmol) was deacetylated according to general method B. The crude product was purified by flash column chromatography (7:3 CH_3_CN:H_2_O) to give compound **13** (70 mg, 66%) as a colourless syrup. [α]^24^_D_ + 44.8 (*c* 0.31, H_2_O); R*_f_* 0.20 (7:3 CH_3_CN:H_2_O); ^1^H NMR (400 MHz, D_2_O) *δ* = 7.94 (s, 8H, 8 x C*H* triazole), 4.97 (s, 16H, 8 x NC*H*_2_ ethylene glycol), 4.93–4.78 (m, 16H, 8 x C*H*_2_ propargyl), 4.44 (d, *J* = 7.6 Hz, 4H, 4 x H-1), 3.94 (d, *J* = 2.8 Hz, 4H, 4 x H-4), 3.83–3.74 (m, 8H, 4 x H-6a,b), 3.71-3.68 (m, 4H, 4 x H-5), 3.65 (dd, *J* = 3.0 Hz, *J* = 9.8 Hz, 4H, 4 x H-3), 3.57–3.53 (m, 4H, 4 x H-2), 3.34 (s, 8H, 4 x C*H*_2_ pentaerythritol) ppm; ^13^C NMR (100 MHz, D_2_O) *δ* = 125.4 (8C, 8 x *C*H triazole), 102.9 (4C, 4 x C-1), 76.2 (4C, 4 x C-5), 73.7 (4C, 4 x C-3), 71.6 (4C, 4 x C-2), 69.6 (4C, 4 x C-4), 69.2 (4C, 4 x *C*H_2_ pentaerythritol), 62.5 (8C, 8 x *C*H_2_ propargyl), 61.9 (4C, 4 x C-6), 50.9 (8C, 8 x N*C*H_2_ ethylene glycol), 45.5 (1C, Cq pentaerythritol) ppm; MALDI-TOF (positive ion): *m/z* calcd for C_61_H_92_N_24_NaO_28_: 1631.64 [M + Na]^+^ Found: 1631.59.

### 2.3. Lectin PA-IL Production and Purification

PA-IL in a recombinant form was produced and purified as previously described [8]. Briefly, transformed *Escherichia coli* BL21(DE3) cells bearing the plasmid for PA-IL (pET25b_pa1l) were cultured in the LB broth medium containing an appropriate antibiotic at 37 °C. When the culture reached an OD_600_ of ≈ 0.5, protein production was induced by isopropyl 1-thio-β-d-galactopyranoside (IPTG) added to a final concentration of 0.5 mM. Cells were incubated at 30 °C for 3 h, harvested by centrifugation and resuspended in a suitable buffer. Cells were then disintegrated by sonication and the cytosolic fraction containing soluble lectin was separated by centrifugation. PA-IL was then purified by affinity chromatography on a Sepharose 4B (GE Healthcare, Chicago, IL, USA) column, dialyzed and further processed according to the previously published procedures [8]. Freeze-dried PA-IL was stored at −20 °C.

### 2.4. Hemagglutination Inhibition Assay (HIA)

PA-IL was dissolved in the buffer with calcium ions suitable for the activity of the lectin (20 mM Tris/HCl, 150 mM NaCl, 5 mM CaCl_2_, pH = 7.5) to the concentration 0.25 mg mL^−1^. Lectin was mixed with carbohydrate inhibitors serially diluted in the buffer in a 5 µL:5 µL ratio. Therefore, the final (working) concentration of the lectin was 0.125 mg mL^−1^. A total of 10 µL of 20% papain-treated, azid-stabilized red blood cells B^+^ in the buffer was then added, and the mixture was thoroughly mixed and incubated for 5 min at room temperature. After incubation, the mixture was again mixed, transferred to a microscope slide, and examined. The examination was conducted using the Levenhuk D2L NG Digital Microscope (Levenhuk, Tampa, FL, USA). Images were obtained with a Levenhuk D2L digital camera (Levenhuk, Tampa, FL, USA) using the software ToupView for Windows (Version 3.7, Levenhuk, Tampa, FL, USA). The positive (experiment without inhibitor) and negative control (experiment without lectin) were prepared and processed in the same way using the appropriate volume of dissolving buffer instead of the omitted components. The minimal inhibitory concentration (MIC) of the inhibitor able to inhibit hemagglutination was determined and compared with the standard (d-galactose) and the potency of the inhibitor was calculated (MIC of the standard/MIC of the inhibitor).

### 2.5. Surface Plasmon Resonance (SPR)

Surface plasmon resonance (SPR) experiments were performed on a BIAcore T200 instrument (GE Healthcare, Chicago, IL, USA) at 25 °C, using HBST running buffer (10 mM HEPES, 150 mM NaCl, 0.5 mM CaCl_2_, 0.05% Tween 20, pH = 7). A CM5 sensor chip (GE Healthcare, Chicago, IL, USA) covered with a carboxymethylated dextran matrix was used for ligand immobilization. The sensor chip surface was activated with *N*-ethyl-*N*-(3-dimethylaminopropyl)carbodiimide/*N*-hydroxysuccinimide solution and then coated with streptavidin (Sigma-Aldrich, St. Louis, MO, USA) in 10 mM acetate pH = 5.0 according to the manufacturer’s standard protocol to a final response of 7000 RU. Unreacted groups were blocked with 1 M ethanolamine-HCl, pH = 8.5. 0.5 mM biotinylated carbohydrate (1:1 mixture of biotin-α-d-galactoside and biotin-β-d-galactoside for measuring channel and α-l-fucoside for blank channel, all Lectinity, Moskow, Russia) was injected into a particular channel at a flow rate of 5 μL min^−1^.

In the experimental setup, SPR inhibition measurements were carried out simultaneously on both measuring and blank channels at a flow rate of 5 µL min^−1^. Protein PA-IL at a concentration of 45 µM was mixed with various concentrations of inhibitors (0.1–125 µM for studied compounds or 5–5000 µM for d-galactose) and injected onto the sensor chip. The response of the blank channel was subtracted from the response of lectin bound to the galactose-modified surface at equilibrium and plotted against the concentration of inhibitor in order to determine IC_50_ (concentration of inhibitor resulting in 50% inhibition of binding). As IC_50_ is not a constant and depends on the experimental set-up, a parameter called potency was used for characterization. The potency of a tested inhibitor is the ratio of IC_50_ of a chosen standard inhibitor (in this case, d-galactose) and the inhibitor in question. Pure lectin PA-IL was used as a control (0% inhibition).

### 2.6. Isothermal Titration Microcalorimetry (ITC)

All the experiments were performed on MicroCal iTC200 (Malvern Instruments, Malvern, UK) at 25 °C. The freeze-dried lectin PA-IL was dissolved in 0.1 M Tris/HCl, 500 μM CaCl_2_, pH = 7.5, and equilibrated at room temperature for 1 h before ITC measurements. Multivalent galactosylated compounds **4**, **7**, **10** and **13** were diluted in the same buffer and used in 0.25–0.5 mM concentrations. Aliquots of 2 µL of the compounds were added automatically to the 0.078 mM or 0.088 mM PA-IL in the calorimeter cell. Blank experiments (injections of compounds to the buffer) were performed and heat responses were subtracted. Integrated heat effects were analyzed by global fitting of data obtained from three independent titrations for each tested compound by non-linear regression using a single site-binding model (Microcal Origin 7, Malvern Instruments, Malvern, UK). Fitted data yielded the association constant (K_a_) and the enthalpy of binding (ΔH). Other thermodynamic parameters, i.e., changes in free energy (ΔG) and entropy (ΔS), were calculated from the following equation: ΔG = ΔH − TΔS = −RT ln K_a_, where T is the absolute temperature and R = 8.314 J mol^−1^ K^−1^. The monosaccharide d-galactose was used as a control; 4 mM d-galactose was added to the 0.147 mM PA-IL in the calorimeter cell. Two titration experiments were performed and the heat responses were processed and evaluated as mentioned above.

### 2.7. Analytical Ultracentrifugation (AUC)

Analytical ultracentrifugation experiments were performed using ProteomeLab XL-I analytical ultracentrifuge (Beckman Coulter, Indianapolis, IN, USA) equipped with an An-60 Ti rotor. Sedimentation velocity experiments were conducted in titanium double-sector centerpiece cells (Nanolytics Instruments, Potsdam, Germany) loaded with 390 µL of both protein sample and reference solution (PBS buffer, pH = 7.5). PA-IL without the added ligand and PA-IL samples pre-incubated with different concentrations of galactosylated dendrimers **4**, **7**, **10** and **13** and d-galactose (protein:ligand ratios of 1:0.1, 1:1 and 1:10) were analyzed by the sedimentation velocity experiment. All the measurements were performed in PBS buffer with 22 µM PA-IL. Data were collected using absorbance optics at 25 °C at a rotor speed of 48,000 rpm. Scans were collected at 280 nm (or alternatively at 294 nm to decrease the absorbance signal of the samples containing excess of the UV-absorbing ligands **4** and **10**) at 5-min intervals and 0.003 cm spatial resolution in continuous scan mode. The partial specific volume of the protein and the solvent density and viscosity were calculated from the amino acid sequence and buffer composition, respectively, using the software Sednterp (http://bitcwiki.sr.unh.edu). The data were analyzed with the continuous c(s) distribution model implemented in the program Sedfit 15.01b [27]. For the regularization procedure, a confidence level of 0.95 was used. The plots of c(s) distributions were created in GUSSI 1.3.1 [28].

### 2.8. Pseudomonas Aeruginosa Cross-Linking Assay

*Pseudomonas aeruginosa* CCM 3955 (ATCC 27853) cells were inoculated from stock cells stored at −80 °C into a standard LB medium and were grown at 37 °C overnight under orbital shaking. The bacterial culture was kept in the fridge until further use. Prior to the experiment, bacterial cells were centrifuged at 4300× *g* (Eppendorf centrifuge MiniSpin, Eppendorf, Germany) for 2 min and subsequently washed three times with PBS buffer (137 mM NaCl, 2.7 mM KCl, 10 mM Na_2_HPO_4_, 1.8 mM KH_2_PO_4_). Bacterial cells were then diluted in PBS to an optical density OD_600_ = 1.5, which was checked using a spectrophotometer (WPA CO8000 Biovawe Cell Density Metr, Biochrom Ltd., Cambridge, UK). d-galactopyranoside-presenting inhibitors were diluted in ultrapure water to the concentration of 100 mM. Bacterial cells were mixed in a 9:1 ratio with the 10x concentrated selected compounds and gently mixed. The mixture was incubated at room temperature for 10 min and thoroughly mixed again. An amount of 5 µL of the mixture was transferred onto a microscope slide and observed under 100x magnification using an optical microscope (Olympus IX81F-3/IX81S1F-3, Olympus, Tokyo, Japan). As a negative control, bacterial culture mixed with ultrapure water in 9:1 ratio was used. The images were obtained using a phase contrast method and the background of the images was subtracted in GIMP.

### 2.9. Pseudomonas Aeruginosa Adherence Assay

#### 2.9.1. Cell Labelling

Epithelial CuFi-1 cells (an immortalized epithelial cell line derived from cystic fibrosis human lungs, ATCC, Kielpin, Poland) were labelled with PKH67 (Fluorescent Cell Linker Kit, Sigma-Aldrich, St. Louis, MO, USA), a green fluorescent membrane marker, as follows. An aliquot of cell suspension containing 7 × 10^6^ cells was washed with PBS buffer and centrifuged (100× *g* for 5 min). The cell pellet was resuspended in 250 µL of Diluent C (Fluorescent Cell Linker Kit, Sigma-Aldrich, St. Louis, MO, USA). Immediately, an equal volume of 8 µM PKH67 in Diluent C (2 µL of PKH67 in 248 µL of Diluent C) was added to the cell suspension. After 5 min incubation at room temperature with periodic mixing, the staining reaction was stopped by adding an equal volume of 1% Fetal Bovine Serum (FBS, Gibco^TM^ Invitrogen, Paisley, UK) in PBS. After 1 min incubation with FBS, the suspension was centrifuged (100× *g* for 10 min). Finally, the cells were washed three times with Bronchial Epithelial Cell Growth Basal Medium (Lonza, Basel, Switzerland), followed by centrifugation (100× *g* for 5 min).

*Pseudomonas aeruginosa* strain 1763 (University Hospital Motol, Prague, Czech Republic) was stained with PKH26 (Fluorescent Cell Linker Kit, Sigma-Aldrich, St. Louis, MO, USA), a red fluorescent membrane marker. Bacteria were grown in a suspension culture in PS medium (peptone/casein digest) in an Erlenmeyer flask for 14 h. The culture was washed with PBS buffer and centrifuged (13,400× *g* for 5 min). The pellet containing 1.5 × 10^9^ bacteria was resuspended in 125 µL of Diluent C (Fluorescent Cell Linker Kit, Sigma-Aldrich, St. Louis, MO, USA) and an equal volume of 16 µM PKH26 in Diluent C (2 µL of PKH26 in 123 µL of Diluent C) was added to the bacterial suspension. The bacteria/dye suspension was incubated for 30 min at room temperature with periodic mixing. The staining was quenched by adding an equal volume of 1% BSA (Bovine Serum Albumin, Merck, Kenilworth, NJ, USA) in PBS. The suspension was incubated with BSA for 1 min to allow the binding of excess dye and then centrifuged (15,700× *g* for 10 min). Bacteria were washed two times with PBS, followed by centrifugation (13,400× *g* for 7.5 and 5 min).

#### 2.9.2. Inhibition of *Pseudomonas Aeruginosa* Adhesion to Epithelial Cells

Epithelial cells CuFi-1 stained with PKH67 were seeded at 8.4 × 10^4^ cells/well onto 96-well plates (CellBIND^®^ 96-well Flat Clear Bottom Black Polystyrene Microplates, Corning Incorporated, Corning, NY, USA) and incubated for 42–45 h at 37 °C, 5% CO_2_ to form a confluent monolayer and regenerate. Prior to the assay, wells were washed with PBS. Compounds **4**, **7**, **10** and **13** were serially diluted with PBS to the desired concentrations. PKH26-stained bacteria *P. aeruginosa* were added to the diluted inhibitor solutions. Bacterial suspensions were immediately added to the wells (50 µL/well). The input ratio was about 100 bacteria per epithelial cell (8.4 × 10^6^ bacteria/well). A suspension without any inhibitor was used as a control. After 2 h incubation at room temperature, the wells were extensively washed three times with PBS to remove non-adhered bacteria. The fluorescence of adhered *P. aeruginosa* cells (Ex 522 nm, Em 569 nm for PKH26) on epithelial cells (Ex 470 nm, Em 505 for PKH67) was quantified using the spectrofluorometer Tecan Infinite M200 Pro (Tecan Group Ltd., Männedorf, Switzerland). Results were expressed as a relative fluorescence ratio of *P. aeruginosa*/CuFi-1. Three independent incubations were performed.

**Figure 1:**
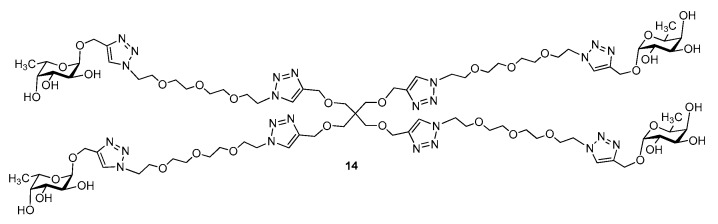
The tetravalent, α-l-fucoside-containing inhibitor with tetraethylene glycol bridges (compound **14**) [29].

The effect of compounds **10** and **13** in combination with previously published tetravalent α-l-fucose-presenting glycocluster (compound **14**, see Figure 1) [29] was also determined. Compounds **10** and **13**, respectively, were serially diluted and mixed with compound **14** in a constant concentration of 0.25 mM. PKH26-stained bacteria *P. aeruginosa* were added to the inhibitors solutions and experiments were performed and evaluated as mentioned above. In addition to control (suspension without any inhibitor), the bacterial suspension with only 0.25 mM compound **14** was also processed and evaluated. Data were analyzed in GraphPad Prism 7 (GraphPad Software, Inc., San Diego, CA, USA).

## 3. Results

### 3.1. Synthesis of Multivalent Galactosides

**Scheme 1:**
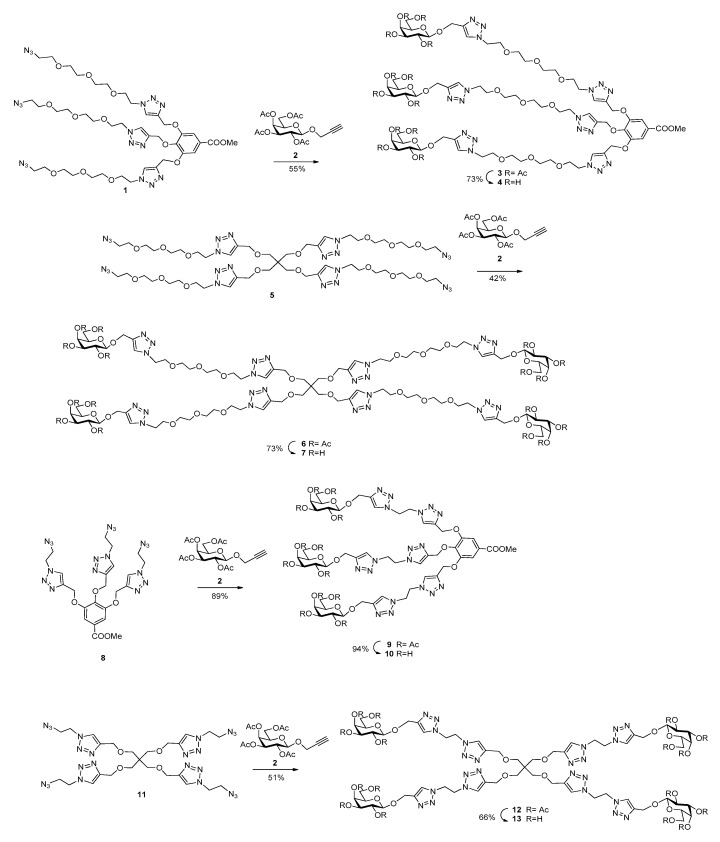
Synthesis of multivalent galactosides.

Similarly to our recent studies [25,30,31], metyl gallate equipped with azide-containing tetraethylene glycol **1** and ethylene glycol chains **8** and pentaerythritol cores **5** and **11** [25] were chosen as multivalent scaffolds (Scheme 1). We efficiently prepared peracetylated propargyl β-d-galactopyranoside **2** by known method [32]. The galactoclusters were built from the above structural elements by applying 1,3-dipolar azide-alkyne cycloaddition click reactions. First, the trivalent cores **1** and **8** were coupled with propargyl 2,3,4,6-tetra-*O*-acetyl-β-d-galactopyranoside **2** via the Cu(I)-catalyzed azide-alkyne cycloaddition click reaction (CuAAC) to get protected trivalent galactosides **3** and **9**, in good yields. Similarly, tetravalent scaffolds **5** and **11** were coupled by CuAAC reaction with galactoside **2** to produce the protected galactoclusters **6** and **12**, respectively. The final products were obtained by Zemplén-deacetylation of **3**, **6**, **9** and **12**, to result in compounds **4**, **7**, **10** and **13**, respectively, showing differences in the valency, flexibility and spatial arrangement of galactosides. Compounds were tested as potential ligands of tetrameric, galactose-specific lectin PA-IL.

### 3.2. Inhibition of PA-IL with Multivalent Galactosylated Compounds

**Figure 2:**
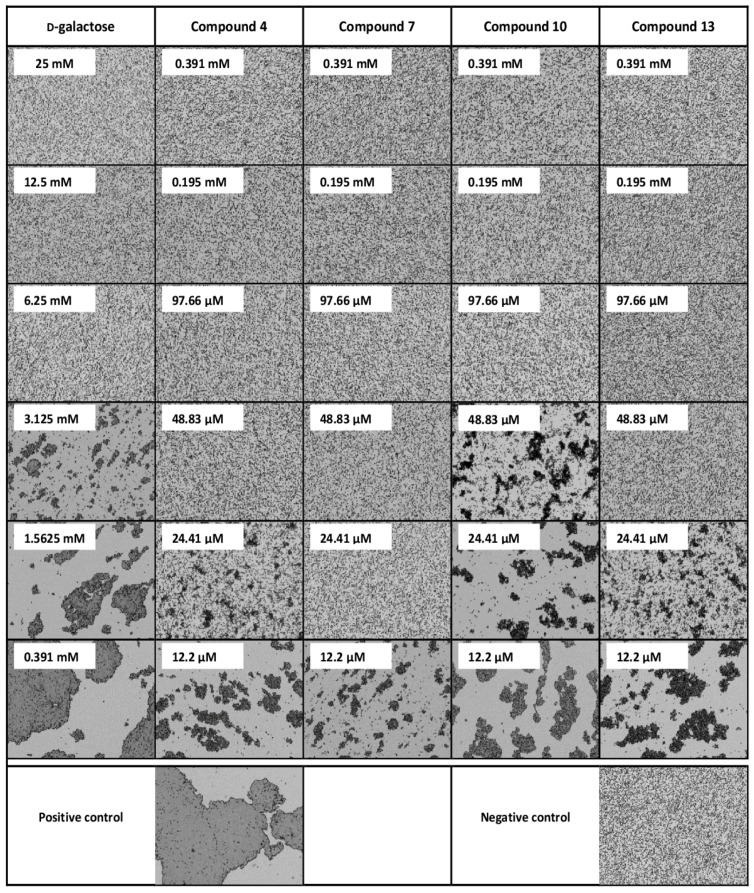
Influence of d-galactose, compounds **4**, **7**, **10**, and **13** on hemagglutination caused by lectin PA-IL. Positive control: experiment without inhibitor. Negative control: experiment without lectin PA-IL.

The preliminary investigations of the interactions of the multivalent galactosylated compounds with PA-IL were performed using hemagglutination inhibition assay with microscope detection. The minimal inhibitory concentrations (MICs) of compounds able to completely inhibit hemagglutination caused by lectin PA-IL were determined and the potencies of the inhibitors were calculated by comparison with the standard (d-galactose). All compounds were able to inhibit hemagglutination significantly better than simple d-galactose (see Table 1 and Figure 2) with compound **7** being the best inhibitor—256 times better than d-galactose. The least efficient inhibitor was compound **10** (with potency value of 64), whereas compounds **4** and **13** were in between (128). Taking into account the simple effect of the increased concentration of galactose units in the multivalent compound (parameter β, potency/valency), compound **7** (the tetravalent compound with longer linkers, β = 64) is still the best inhibitor and compound **10** (trivalent with shorter ethylene-linker, β = 21.3) the least efficient. Considering compounds **4** and **13**, differences in inhibitory efficiency were observed. Compound **4** (trivalent, tetraethylene glycol-linker, β = 42.7) was better than compound **13** (tetravalent, ethylene-linker, β = 32). Generally, a more efficient inhibitory activity was associated with tetravalent compounds and longer linkers.

**Figure 3:**
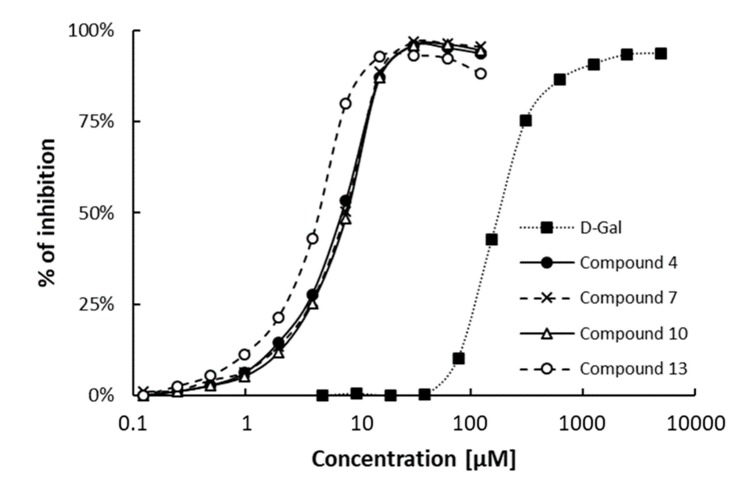
Inhibitory effects of tested compounds on lectin PA-IL binding to immobilized d-galactosides obtained by SPR. Individual type of line corresponds to monovalent (dotted), trivalent (solid) and tetravalent (dashed) ligands. d-galactose was used as a control and standard.

The surface plasmon resonance (SPR) technique was employed to further analyse the competitive inhibition of PA-IL binding to a multivalent chip surface. Sensor chip presenting d-galactopyranoside residues was treated by a constant concentration of PA-IL, and the concentrations of the tested inhibitors were gradually decreased to determine IC_50_. All the tested compounds clearly demonstrated a higher inhibition potency when compared to d-galactose (see Table 2). The most potent inhibitor appeared to be compound **13,** with a more than 40 times higher inhibition potency than d-galactose (more than 10 times when calculating parameter β considering the effect per monomer unit) followed by compounds **4**, **10** and **7**, that displayed almost identical inhibitory effects (see Figure 3).

### 3.3. Thermodynamics of the Interactions

The interactions of PA-IL with d-galactopyranoside-presenting glycoclusters were directly characterized by isothermal titration calorimetry. All the compounds were able to bind to PA-IL with an affinity three orders of magnitude higher than simple d-galactose (see Table 3). Interactions were exothermic- and enthalpy-driven, with significantly unfavourable entropy. Interactions with tetravalent compounds displayed higher unfavourable entropy values than those with trivalent compounds, probably corresponding to the larger loss of flexibility during binding. However, the entropy loss was connected with increased favourable enthalpy values in an example of enthalpy-entropy compensation [33]. The best binding partner for lectin PA-IL, according to the obtained affinity constants, was compound **13**, followed by compound **7,** i.e., the tetravalent variants. The stoichiometry (n value) of interactions was lower than 1, suggesting that one molecule of inhibitor is recognized by several binding sites of lectin PA-IL. No aggregation was observed during or after the calorimetry experiments, implying that the binding is either intramolecular (within the same PA-IL tetramer) or the particles resulting from intermolecular binding are too small to form visually observable aggregates.

### 3.4. Cross-linking of Lectin PA-IL with Multivalent Galactosylated Inhibitors

**Figure 4:**
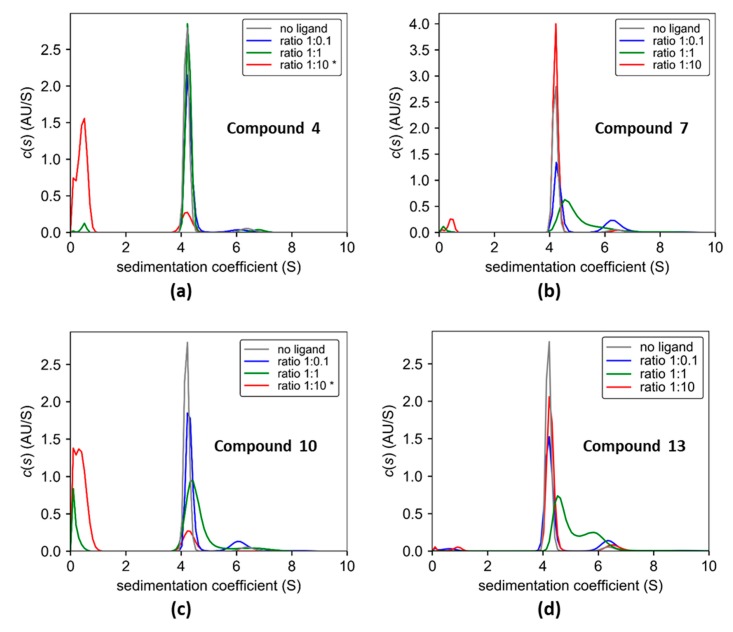
Continuous c(s) distributions of PA-IL samples obtained in the absence and presence of multivalent galactosylated dendrimers. The distributions were obtained at different protein to ligand ratios (see the legend). The c(s) curves obtained at 294 nm (instead of 280 nm) are marked with a star. (**a**) Results for compound **4**; (**b**) Results for compound **7**; (**c**) Results for compound **10**; (**d**) Results for compound **13**.

The sedimentation velocity experiment was used to investigate the cross-linking properties of the galactosylated dendrimers (compounds **4**, **7**, **10** and **13**) on the lectin PA-IL. In the absence of dendrimers, PA-IL was mostly present as a tetramer with the sedimentation coefficient of 4.2 S (estimated molar mass of 48.4 kDa), about 5% of PA-IL had an s-value of 6.4 S and corresponded most likely to an octameric species. The addition of dendrimers **7**, **10** and **13** (but not **4**) induced the formation of the 6.4 S particle (see Figure 4). At a 1:0.1 protein to ligand ratio, the highest level of cross-linking was observed for compound **7** (~30%), followed by compounds **10** and **13** (~15%). In the molar excess of the ligand, the percentage of 6.4 S species decreased and was comparable to the sample without the ligand added. The ligands (if absorbing in UV) had a sedimentation coefficient between 0.3 and 0.5 S. Since the formation of larger oligomers or precipitation were not detected by analytical ultracentrifugation, the data suggests that dendrimers **7**, **10** and **13** cross-link PA-IL in a well defined way (formation of octamers). In the control experiment, d-galactose had a negligible effect on the percentage of the 6.4 S particle (see Figure S1). According to the results, trivalent compound **4** did not form intermolecular cross-links in the used set-up, probably due to the combination of the topology of its branches and the length of its linkers. Compared with the trivalent compound **10**, which forms intermolecular cross-links, the longer linkers of compound **4** are probably more suitable for intramolecular binding to several binding sites of PA-IL. In the molar excess of all the compounds, the binding sites of PA-IL seemingly bind separate molecules of dendrimers, which prevents cross-linking.

### 3.5. Cross-linking of Pseudomonas Aeruginosa Cells with Multivalent Galactosylated Inhibitors

**Figure 5:**
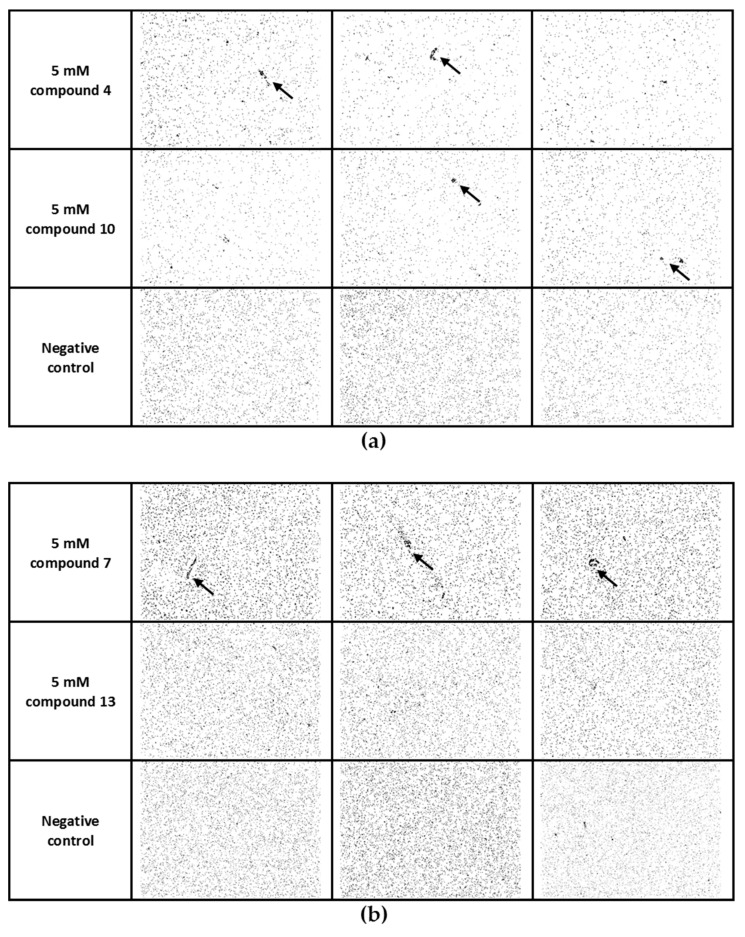
Cross-linking of *Pseudomonas aeruginosa* cells with d-galactopyranoside-presenting inhibitors. The *P. aeruginosa* cells with no added inhibitors were used as a control. Magnification 100x, phase contrast, background subtraction in GIMP, triplicates. The results for the highest concentrations used are presented. For the other concentrations used, see Figures S2 and S3 in the Supplementary data. The representative aggregates are highlighted by the black arrows. (**a**) Results for compounds **4** and **10**; (**b**) Results for compounds **7** and **13**.

The abilities of d-galactopyranoside-presenting inhibitors to cross-link whole *Pseudomonas aeruginosa* cells via interactions with lectin PA-IL on the surface were investigated by cell cross-linking assay with microscope detection. The cell aggregates clearly distinguishable from the negative control were only observed in low quantities and mainly in highly used concentrations of the tested compounds (2.5–5 mM, see Figure 5). The cell cross-linking activity of the tested compounds was either generally weak or the resulting cell aggregates were too small to be detected by the used methodology in the current set-up. Considering compound **13**, no aggregates were observed, even in 5 mM concentration. On the other hand, compound **7** displayed the highest cross-linking activity and aggregates distinguishable from the negative control were observed even in a concentration of 1.25 mM (see Figure S2).

### 3.6. Inhibition of Pseudomonas Aeruginosa Adhesion to Epithelial Bronchial Cells

**Figure 6:**
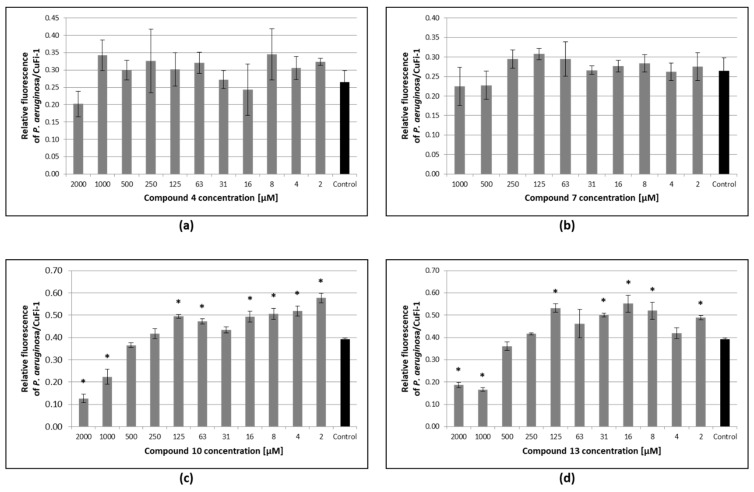
Adherence of *P. aeruginosa* to human bronchial epithelial cells (CuFi-1) in the presence of the tested compounds. Monolayers of PKH67-labeled CuFi-1 cells were incubated for 2 h with suspensions containing PKH26-labeled *P. aeruginosa* and tested compounds (grey bars) or PBS as a control (black bars). After discarding of non-adhered bacteria, the relative fluorescence of adhered *P. aeruginosa* and CuFi-1 was quantified using Tecan Infinite M200 Pro. Results are expressed as a relative fluorescence ratio of *P. aeruginosa*/CuFi-1 plotted as a function of compounds concentrations. Plotted data are means ± SD of three independent incubations. * indicates significant difference (*p* < 0.05; Dunnett test). (**a**) Results for compound **4**; (**b**) Results for compound **7**; (**c**) Results for compound **10**; (**d**) Results for compound **13**.

**Figure 7:**
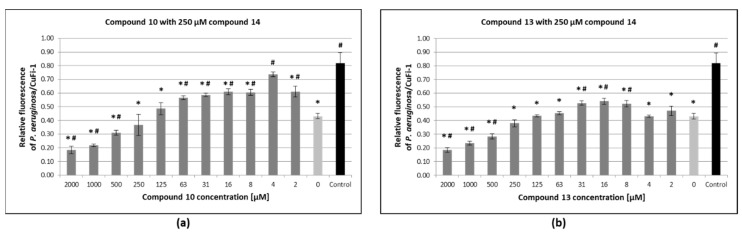
Adherence of *P. aeruginosa* on CuFi-1cells in the presence of compounds combination. Monolayers of PKH67-labeled CuFi-1 cells were incubated for 2 h with suspensions containing PKH26-labeled *P. aeruginosa* and 250 μM compound **14** combined with compound **10** or **13** (dark grey bars) or only 250 μM compound **14** (light grey bar). Suspension with PKH26-labeled *P. aeruginosa* in PBS was used as an untreated control (black bar). After discarding non-adhered bacteria, the relative fluorescence of adhered *P. aeruginosa* and CuFi-1 was quantified using Tecan Infinite M200 Pro. Results are expressed as a relative fluorescence ratio of *P. aeruginosa*/CuFi-1 plotted as a function of anti-PA-IL compounds concentration. Plotted data are means ± SD of three independent incubations. * indicates significant difference from untreated control (*p* < 0.05; Dunnett test). ^#^ indicates significant difference from experiment using only 250 μM compound **14** (*p* < 0.05; Dunnett test). (**a**) Results for compound **10**; (**b**) Results for compound **13**.

Ex vivo bacterial adherence assay was used for the evaluation of the ability of tested compounds to inhibit the adhesion of *P. aeruginosa* (ST 1763, isolated from a CF patient) to epithelial bronchial cells (CuFi-1, derived from a CF patient). Bacteria labeled with PKH26 were incubated with PKH67-labeled CuFi-1 in the presence of compounds **4**, **7**, **10** and **13** or PBS as untreated control (see Figure 6). Compounds **4** and **7** displayed no inhibitory effect on bacterial adhesion compared to the control. On the contrary, compounds **10** and **13** had a significant protecting effect in concentrations ≥1 mM. In the highest concentration (2 mM), compounds **10** and **13** were able to reduce bacterial adhesion to approximately 32% and 42%, respectively, compared to the untreated control. However, the addition of compounds **10** and **13** in some lower concentrations (≤125 μM) resulted in a statistically significantly increased fluorescent signal (see Figure 6c,d). Furthermore, the combined effect of anti-PA-IL inhibitors (compounds **10** and **13**) and the previously published PA-IIL inhibitor (compound **14**) was tested (see Figure 7). PA-IIL (LecB) is another lectin from *Pseudomonas aeruginosa* involved in pathogenicity [8]. According to the previous study, compound **14** was used in its most effective concentration (250 μM) [29] and combined with the concentration gradient of compound **10** or **13.** The mixtures of compounds **10** or **13** in the highest concentration (2 mM) with compound **14** (250 μM) reduced the *P. aeruginosa* adhesion to approximately 22% and 23%, respectively. Considering these combined experiments, no response exceeding the untreated control was observed for any used concentration of compounds **10** and **13**.

## 4. Discussion

An expeditious click-chemistry approach was applied to synthesize tri- and tetravalent glycodendrimers of different flexibilities bearing the β-d-galactoside units with different spatial arrangements. The ability of the synthetized β-d-galactopyranoside-containing tri- and tetravalent glycoclusters to inhibit the lectin PA-IL (LecA) from *Pseudomonas aeruginosa* was determined by hemagglutination inhibition assay (HIA) with microscope detection and by SPR inhibition assay using immobilized d-galactopyranoside. Hemagglutination inhibition assay is a quick and robust method commonly used for characterization of lectin-carbohydrate interactions [34]. The carbohydrate-covered surface of red blood cells enables lectins to employ their multivalency and potential inhibitors could be suitably tested for their ability to compete with this native surface. On the other hand, hemagglutination assay relies fully on visual detection and thorough comparisons with positives and negatives controls. SPR inhibition assay in flow-through arrangement with carbohydrates immobilized on the static sensor chip could theoretically better mimic the real environment of lectin’s action—binding to lung tissues. However, SPR is sensitive to aggregation and potential artifacts caused by cross-linking of lectins [30]. By combination of these two diverse methods, their weaknesses could be compensated and the inhibitory activity of tested compounds verified. According to the hemagglutination assay, tetravalent compound **7** was the best inhibitor (potency 256, β = 64). However, the trivalent compound **4** (potency 128, β = 42.7) was the second best compound, suggesting a preference for compounds with longer and more flexible tetraethylene glycol-linker over shorter ethylene-linker, regardless of their valency. Considering SPR assay, compound **13** was determined to be the best inhibitor. In contrast to hemagglutination assay, there was no clear correlation observed between the compounds’ valency or structure and their inhibitory effect. This demonstrates not only the importance of structural arrangement of the studied compounds but also of the nature of the tested surface. As SPR and HIA have completely different set-ups and detection principles, the acquisition of different data was not surprising. The fact that diverse techniques for determination of inhibitory potency provide different and sometimes even contradictory results is well known [11,35] and hinders the reliable in vitro evaluation of inhibitors. Nevertheless, both methods confirmed that all the tested compounds could inhibit PA-IL significantly better than simple d-galactose.

**Figure 8:**
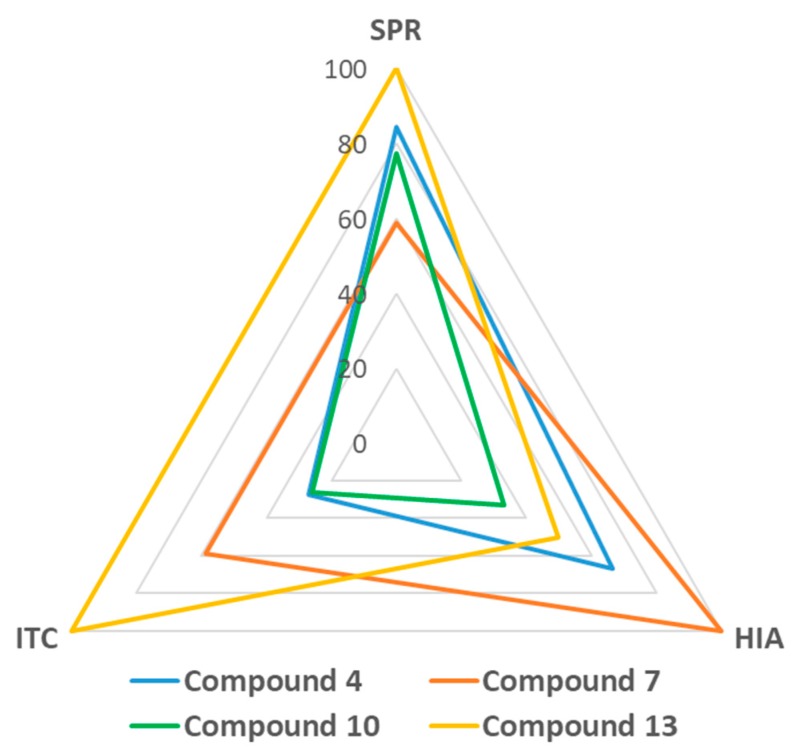
Comparison of effectiveness of tested compounds obtained by hemagglutination inhibition assay (HIA), SPR inhibition assay (SPR) and isothermal titration microcalorimetry (ITC). Values for parameters β (HIA and SPR) and K_a_ (ITC) were relativized to the best inhibitor/ligand (100%) obtained by each method and compared.

The interactions of β-d-galactopyranoside-containing compounds and PA-IL were also characterized by isothermal titration microcalorimetry to obtain true binding affinities and thermodynamic parameters. Interactions were enthalpy-driven with strong unfavorable entropy. As carbohydrates are very flexible molecules, a strong entropy barrier is usually observed during binding, i.e., fixation of carbohydrates into the binding sites [36]. Binding affinities were three orders of magnitude higher than for d-galactose. The affinities obtained by ITC are not equal to inhibitory activities but could correlate with them. The best ligand for the lectin PA-IL in the ITC set-up (in solution method) was compound **13**, followed by compound **7**, i.e., the tetravalent ligands. Although the entropy loss was significantly higher during interactions with tetravalent compounds than with trivalent, the enthalpy compensation resulted in higher affinities to tetravalent variants. The combination of inhibition assays and direct affinity measurement confirms that the tested compounds are suitable ligands/inhibitors of lectin PA-IL but the variation of the results (see Figure 8) obtained by different methods thwarted the straightforward selection of compounds with the highest potential to affect *Pseudomonas aeruginosa* in vivo.

The PA-IL-cross-linking ability of carbohydrate-based inhibitors was associated with their potential to inhibit *Pseudomonas aeruginosa* biofilm formation [23]. Moreover, to determine the possible correlation between cross-linking and anti-adhesion properties, analytical ultracentrifugation with lectin PA-IL and cross-linking assay with whole *P. aeruginosa* cells were employed. Considering AUC, compounds **7**, **10** and **13** cross-linked PA-IL in well defined manner, forming octamers. The formation of octamers decreased in the molar excess of tested compounds, suggesting saturation of binding sites of PA-IL. Compound **7** was the best cross-linker; on the other hand, no cross-linking activity was observed for compound **4**. The cross-linking assay with whole cells was further used to better mimic the in vivo condition. Formation of aggregates was observed for compounds **7**, **10** and, in contrast to AUC, also for compound **4**. On the other hand, no clearly distinguishable aggregates were detected when using compound **13**. Generally, the number of aggregates was low, suggesting a low cross-linking activity in the used set-up or the formation of aggregates too small to be reliably detected by the utilized methodology.

The contradictory results from inhibition/affinity assays and cross-linking assays demonstrates the problems with the in vitro evaluation of potential inhibitors. Since the in vivo testing (especially for potential human drugs) is beyond the possibility of basic research, the ex vivo bacterial adherence assay was chosen as the closest possible to the real system. This assay was used to monitor the effect of the tested compounds on bacteria–cell interaction, utilizing the epithelial cells and *P. aeruginosa* strain derived from a cystic fibrosis (CF) patient and direct detection of adhered bacteria by fluorescence. Although compounds **4** and **7** performed well in in vitro tests, they have essentially no visible protective effect in the adhesion assay. On the other hand, compounds **10** and **13** displayed significant anti-adhesion effect in concentrations ≥1 mM and could be suitable for prophylaxis against the bacterial colonization of bronchial cells. However, the efficient concentrations were rather high, which could be difficult to achieve in vivo and could increase the possibility of negative side effects. Furthermore, the increase of fluorescence signal (i.e., the number of adhered bacterial cells) was observed in lower concentrations (≤125 μM) of compounds. This could have been caused by the cross-linking of PA-IL on the surface of the bacterial cells and corresponds to the results from AUC assay. Alternatively, some galactose-binding protein on the surface of epithelial cells could additionally interact with β-d-galactopyranoside-containing compounds, linking bacteria and epithelial cells together. The insufficient maintenance of effective concentration in the lungs of CF patients could theoretically lead to an *increased* adhesion of *P. aeruginosa* to the tissues. Combined with the fact that compound **7** (the best cross-linker) failed in the adherence assay, the cross-linking ability cannot be associated with anti-adhesion properties.

In addition to PA-IL, *Pseudomonas aeruginosa* also produces a fucose-specific lectin PA-IIL (LecB), which is involved in the adhesion and in the biofilm formation [37] and was shown to block ciliary beating of epithelial cells [8]. As PA-IIL has a completely different carbohydrate specificity than PA-IL, the β-d-galactopyranoside-containing compounds cannot inhibit this lectin and combination of specific inhibitors is necessary for maximal inhibition of adhesion. Therefore, the combined effect of compounds **10** or **13,** respectively, and previously prepared tetravalent α-l-fucose-presenting glycocluster **14** [29] was evaluated. The most effective combinations of inhibitors (2 mM compound **10** or **13** plus 250 μM compound **14**) were able to reduce bacterial adhesion to approximately 22% and 23%, respectively. The increase of fluorescent signal in lower concentrations of compounds **10** and **13** (compared to the experiment using only 250 μM compound **14**), was observed in these combined experiments, too. However, it did not exceed the value of untreated negative control. Therefore, we suggest the use of a combination of galactose/fucose-containing inhibitors for prophylaxis purposes to achieve maximal anti-adhesion activity and the elimination of possible negative effects caused by using only galactose-based inhibitors. Considering CF patients, the problem of highly effective concentrations of tested compounds could be overcome by the fact that it is feasible to reach high doses of medication applied directly to lungs via inhalation [38,39].

## 5. Conclusions

β-d-galactopyranoside-containing tri- and tetravalent glycoclusters, synthesized by click-chemistry, were tested by a variety of methods to determine their inhibitory effect against the lectin PA-IL from *Pseudomonas aeruginosa.* All the compounds were found to be suitable inhibitors/ligands of the lectin in vitro, with a significantly better inhibitory effect/affinity than simple d-galactose. Compounds **10** and **13** were also able to decrease adhesion of *P. aeruginosa* cells to bronchial human cells in the ex vivo adhesion assay. In contrast to inhibition of biofilm formation [23], the cross-linking ability of multivalent inhibitors could not be directly associated with the anti-adhesion potential. The best results in adhesion assay were obtained for compounds **10** and **13** in combination with previously investigated tetravalent α-l-fucose-containing inhibitor of lectin PA-IIL, another virulence factor of *P. aeruginosa.* Therefore, future research will be focused on the simultaneous inhibition of both lectins, possibly by using mixed fucose/galactose-based inhibitors. In conclusion, tri- and a tetravalent compounds **10** and **13** are promising candidates for testing using a mouse cystic fibrosis model [40] with potential future utilization as prophylactic agents against bacterial colonization of lungs.

## Figures and Tables

**Table 1:** The MIC (minimal inhibitory concentration) values and potencies of tested inhibitors obtained for the inhibition of hemagglutination caused by lectin PA-IL.

Inhibitor	MIC	Potency ^2^	Valency	β ^3^
d-galactose ^1^	6.25 mM	1	1	1
Compound **4**	48.83 µM	128	3	42.7
Compound **7**	24.41 µM	256	4	64
Compound **10**	97.66 µM	64	3	21.3
Compound **13**	48.83 µM	128	4	32

^1^ Standard, ^2^ MIC of standard/MIC of inhibitor, ^3^ Potency/Valency.

**Table 2:** The IC_50_ (concentration of inhibitor resulting in 50% inhibition of binding) values and potencies of the tested inhibitors obtained by SPR for lectin PA-IL.

Inhibitor	IC_50_	Potency ^2^	Valency	β ^3^
d-galactose ^1^	187.1 µM	1	1	1
Compound **4**	6.8 µM	27.5	3	9.2
Compound **7**	7.3 µM	25.6	4	6.4
Compound **10**	7.4 µM	25.3	3	8.4
Compound **13**	4.3 µM	43.5	4	10.9

^1^ Standard, ^2^ IC_50_ of standard/IC_50_ of inhibitor, ^3^ Potency/Valency.

**Table 3:** Thermodynamic parameters of the interactions between lectin PA-IL and the tested inhibitors.

Inhibitor	Valency	*n*	K_a_ [10^4^ M^−1^]
d-galactose	1	0.78 ± 0.05	0.68 ± 0.038
Compound **4**	3	0.51 ± 0.01	128 ± 15.1
Compound **7**	4	0.32 ± 0.01	281 ± 23.5
Compound **10**	3	0.53 ± 0.01	122 ± 12.8
Compound **13**	4	0.21 ± 0.01	470 ± 54.5
**Inhibitor**	**ΔH [kJ/mol]**	**−T** **ΔS [kJ/mol]**	**ΔG [kJ/mol]**
d-galactose	−37.8 ± 2.92	15.9 ± 2.12	−21.9 ± 1.23
Compound **4**	−80.7 ± 1.01	45.9 ± 5.99	−34.8 ± 4.11
Compound **7**	−110.0 ± 0.82	72.7 ± 6.65	−36.8 ± 3.09
Compound **10**	−68.2 ± 0.75	33.4 ± 3.87	−34.7 ± 3.64
Compound **13**	−134.0 ± 1.45	95.7 ± 12.15	−38.1 ± 4.42

## Supplementary Materials

The following are available online at https://www.mdpi.com/2218-273X/9/11/686/s1, Figure S1: Continuous c(s) distributions of PA-IL samples obtained in the absence and presence of d-galactose. Figure S2: Cross-linking of *Pseudomonas aeruginosa* cells with d-galactopyranoside-presenting inhibitors. Results for compounds **7** and **13**. Figure S3: Cross-linking of *Pseudomonas aeruginosa* cells with d-galactopyranoside-presenting inhibitors. Results for compounds **4** and **10**. Figure S4: ^1^H, ^13^C NMR, COSY and HSQC spectra of compound **4**. Figure S5: ^1^H, ^13^C NMR, COSY and HSQC spectra of compound **7**. Figure S6: ^1^H, ^13^C NMR, COSY and HSQC spectra of compound **10**. Figure S7: ^1^H, ^13^C NMR, COSY and HSQC spectra of compound **13**.
