# Supplementary material for: Synthesis of β-d-galactopyranoside-Presenting Glycoclusters, Investigation of Their Interactions with Pseudomonas aeruginosa Lectin A (PA-IL) and Evaluation of Their Anti-Adhesion Potential
Source: Biomolecules. 2019 Nov 1;9(11):686. doi: 10.3390/biom9110686 (PMC6920806; doi:10.3390/biom9110686)
Supplement: Supplementary file 1 [file biomolecules-09-00686-s001.pdf]

Synthesis of β-D-galactopyranoside-presenting glycoclusters, investigation of their interactions with *Pseudomonas aeruginosa* lectin A (PA-IL) and evaluation of their anti-adhesion potential

Lenka Malinovská [1,2], Son Thai Le [3,4], Mihály Herczeg [3,5], Michaela Vašková [5], Josef Houser [1,2], Eva Fujdiarová [2], Jan Komárek [1,2], Petr Hodek [6], Anikó Borbás [3], Michaela Wimmerová [1,2,7,]* and Magdolna Csávás [3,]*

[1] Central European Institute of Technology, Masaryk University, Kamenice 5, 625 00 Brno, Czech Republic; malinovska@mail.muni.cz (L.M.), houser@mail.muni.cz (J.H.), honzakomarek@mail.muni.cz (J.K.), michaw@chemi.muni.cz (M.W.)

[2] National Centre for Biomolecular Research, Faculty of Science, Masaryk University, Kotlářská 2, 611 37 Brno, Czech Republic; eva.fujdiarova@mail.muni.cz (E.F.)

[3] Department of Pharmaceutical Chemistry, University of Debrecen, Egyetem tér 1, H-4032, Debrecen, Hungary; le.thai.son@pharm.unideb.hu (S.L.T.), herczeg.mihaly@science.unideb.hu (M.H.), borbas.aniko@pharm.unideb.hu (A.B.), csavas.magdolna@science.unideb.hu (M.C.)

[4] Doctoral School of Pharmaceutical Sciences, University of Debrecen, Egyetem tér 1, H-4032, Debrecen, Hungary

[5] Research Group for Oligosaccharide Chemistry of Hungarian Academy of Sciences, Egyetem tér 1, H-4032, Debrecen, Hungary

[6] Department of Biochemistry, Faculty of Science, Charles University, Albertov 2030, 128 40 Prague 2, Czech Republic; michael.vaskova@gmail.com (M.V.); petr.hodek@natur.cuni.cz (P.H.)

[7] Department of Biochemistry, Faculty of Science, Masaryk University, Kotlářská 2, 611 37 Brno, Czech Republic

* Correspondence: michaw@chemi.muni.cz (M.W.); csavas.magdolna@science.unideb.hu (M.C.); Tel.: +420-549-49-3805 (M.W.); +3652-512900-22472 (M.C.)

Received: ; Accepted: ; Published:

Abstract: *Pseudomonas aeruginosa* is an opportunistic human pathogen associated with cystic fibrosis. This bacterium produces, among other virulence factors, a soluble D-galactose-specific lectin PA-IL (LecA). PA-IL plays an important role in the adhesion to the host cells and is also cytotoxic. Therefore, this protein is an interesting therapeutic target, suitable for inhibition by carbohydrate-based compounds. In the current work, β-D-galactopyranoside-containing tri- and tetravalent glycoclusters were synthesized. Methyl gallate and pentaerythritol equipped with propargyl groups were chosen as multivalent scaffolds and the galactoclusters were built from the above-mentioned cores by coupling ethylene or tetraethylene glycol-bridges and peracetylated propargyl β-D-galactosides using 1,3-dipolar azide-alkyne cycloaddition. The interaction between galactoside derivatives and PA-IL was investigated by several biophysical methods, including hemagglutination inhibition assay, isothermal titration calorimetry, analytical ultracentrifugation, and surface plasmon resonance. Their ability to inhibit adhesion of *P. aeruginosa* to bronchial cells was determined by *ex vivo* assay. The newly synthesized multivalent galactoclusters proved to be significantly better ligands than simple D-galactose for lectin PA-IL and as a result, two representatives of the dendrimers were able to decrease adhesion of *P. aeruginosa* to bronchial cells to approx. 32 % and 42 %, respectively.

Keywords: *Pseudomonas aeruginosa*; cystic fibrosis; lectin; D-galactosides; multivalency

Supplementary data

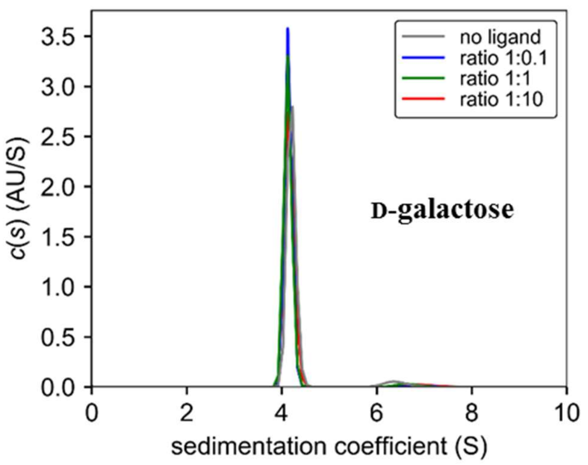

Figure S1. Continuous c(s) distributions of PA-IL samples obtained in the absence and presence of D-galactose. The distributions were obtained at different protein to ligand ratios (see the legend).

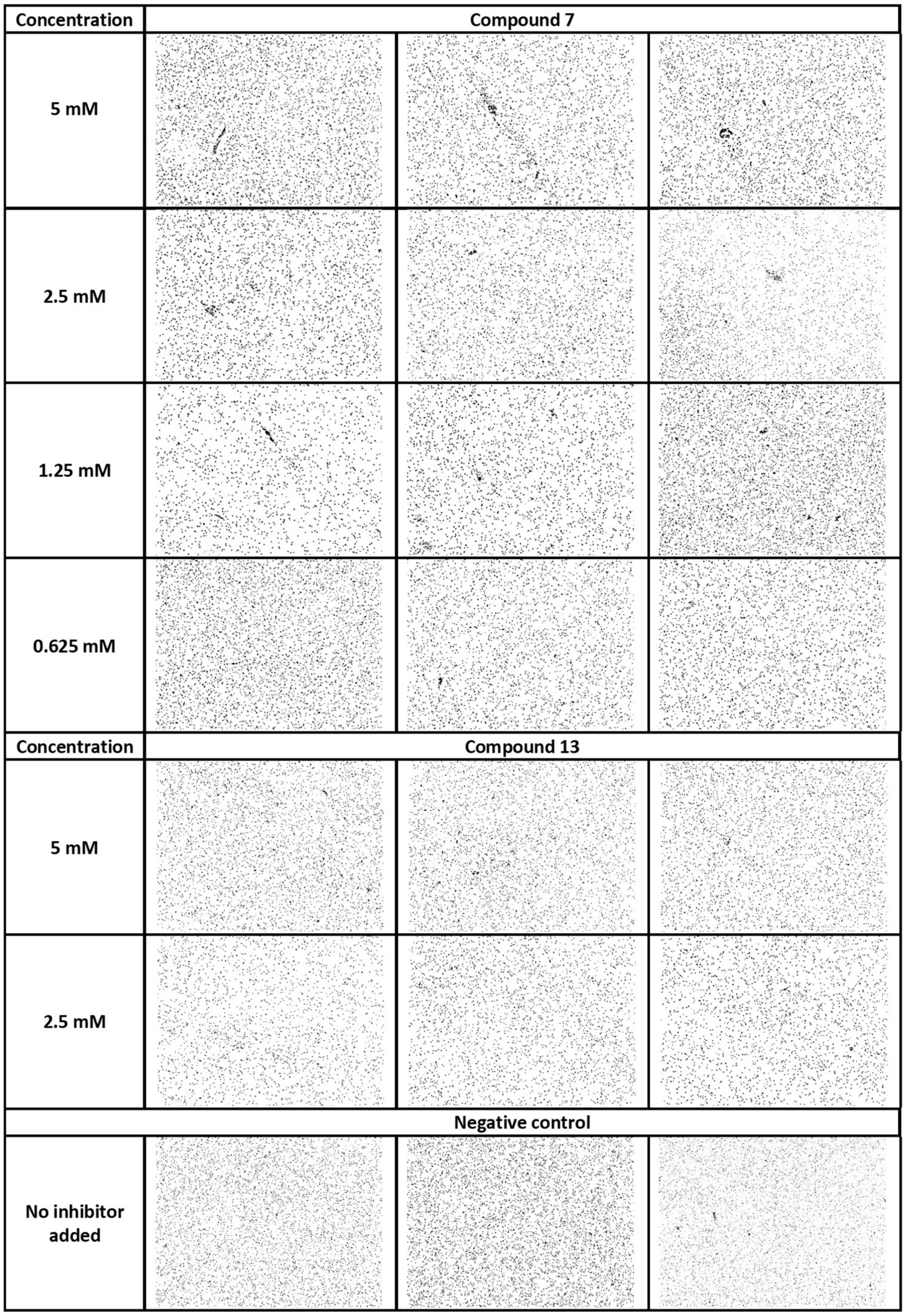

Figure S2. Cross-linking of *Pseudomonas aeruginosa* cells with D-galactopyranoside-presenting inhibitors. The *P. aeruginosa* cells with no added inhibitors were used as a control. Magnification 100x, phase contrast, background subtraction in GIMP, triplicates. Results for compounds **7** and **13**.

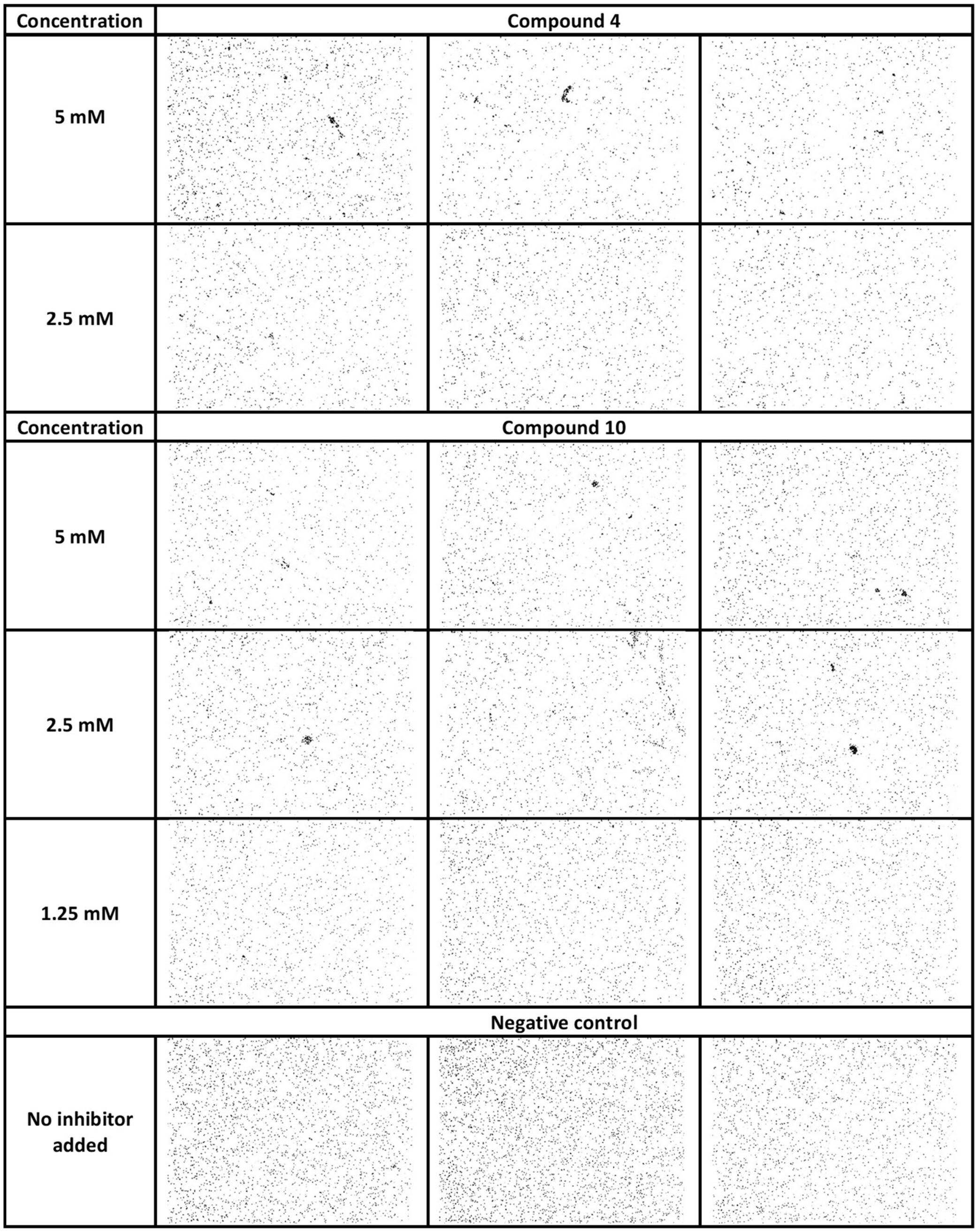

Figure S3. Cross-linking of *Pseudomonas aeruginosa* cells with D-galactopyranoside-presenting inhibitors. The *P. aeruginosa* cells with no added inhibitors were used as a control. Magnification 100x, phase contrast, background subtraction in GIMP, triplicates. Results for compounds **4** and **10**.

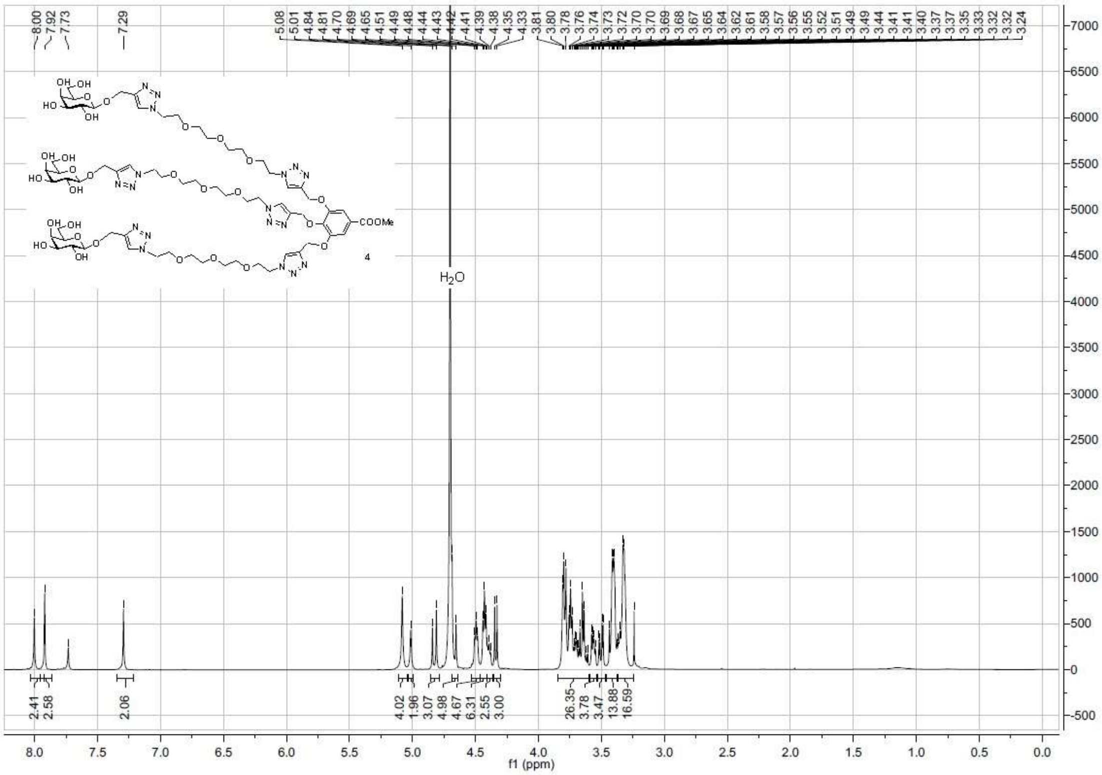

8.00
7.92
7.73
7.29
5.08
5.01
4.84
4.81
4.70
4.69
4.65
4.51
4.49
4.48
4.44
4.43
4.42
4.41
4.39
4.38
4.35
4.33
3.81
3.80
3.78
3.76
3.74
3.73
3.72
3.70
3.70
3.69
3.68
3.67
3.65
3.64
3.62
3.61
3.58
3.57
3.56
3.55
3.52
3.51
3.49
3.49
3.44
3.41
3.41
3.40
3.37
3.35
3.33
3.32
3.32
3.24
COOMe
4
H_2O
2.41
2.58
2.06
4.02
1.96
3.07
4.98
4.67
6.31
2.55
3.00
26.35
3.78
3.47
13.88
16.59
f1 (ppm)

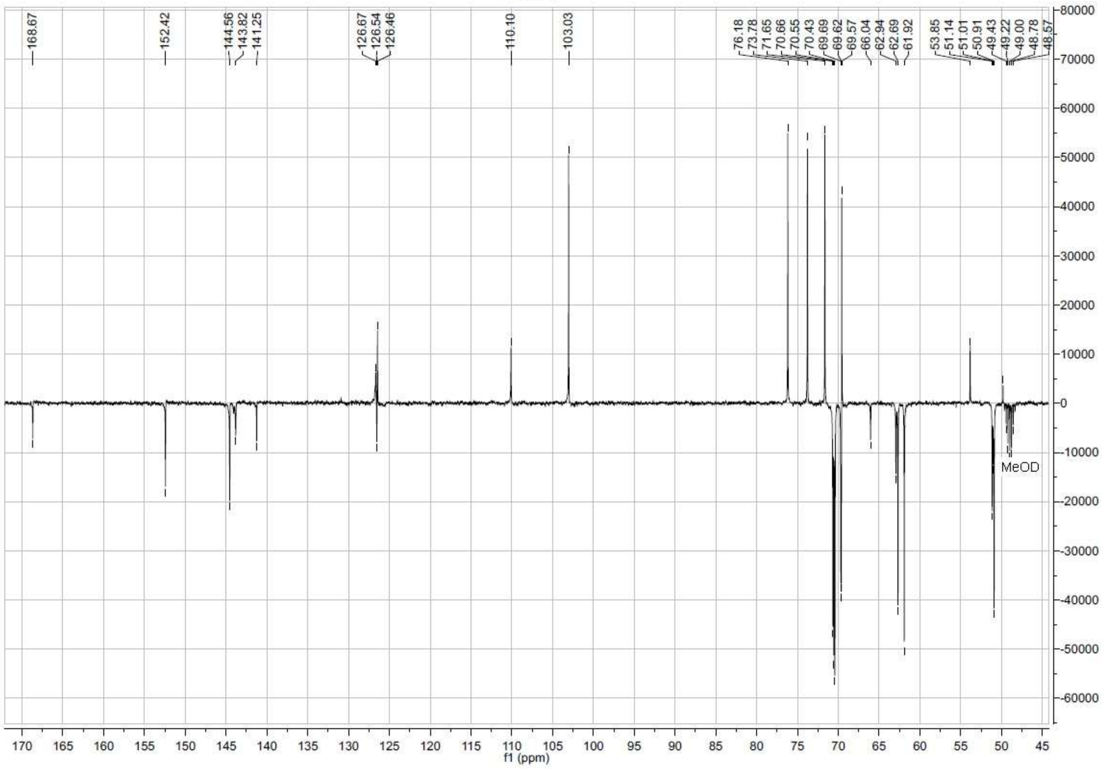

168.67
152.42
144.56
143.82
141.25
126.67
126.54
126.46
110.10
103.03
76.18
73.78
71.65
70.66
70.55
70.43
69.69
69.62
69.57
66.04
62.94
62.69
61.92
53.85
51.14
51.01
50.91
49.43
49.22
49.00
48.78
48.57
MeOD
f1 (ppm)

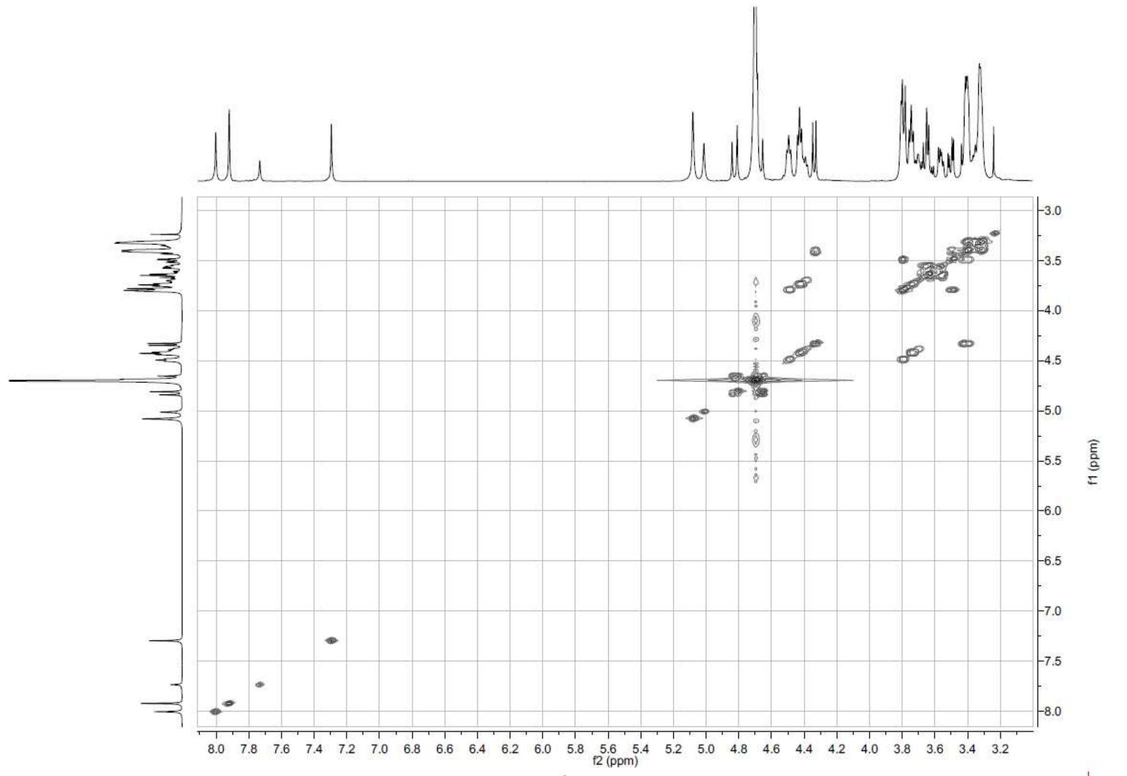

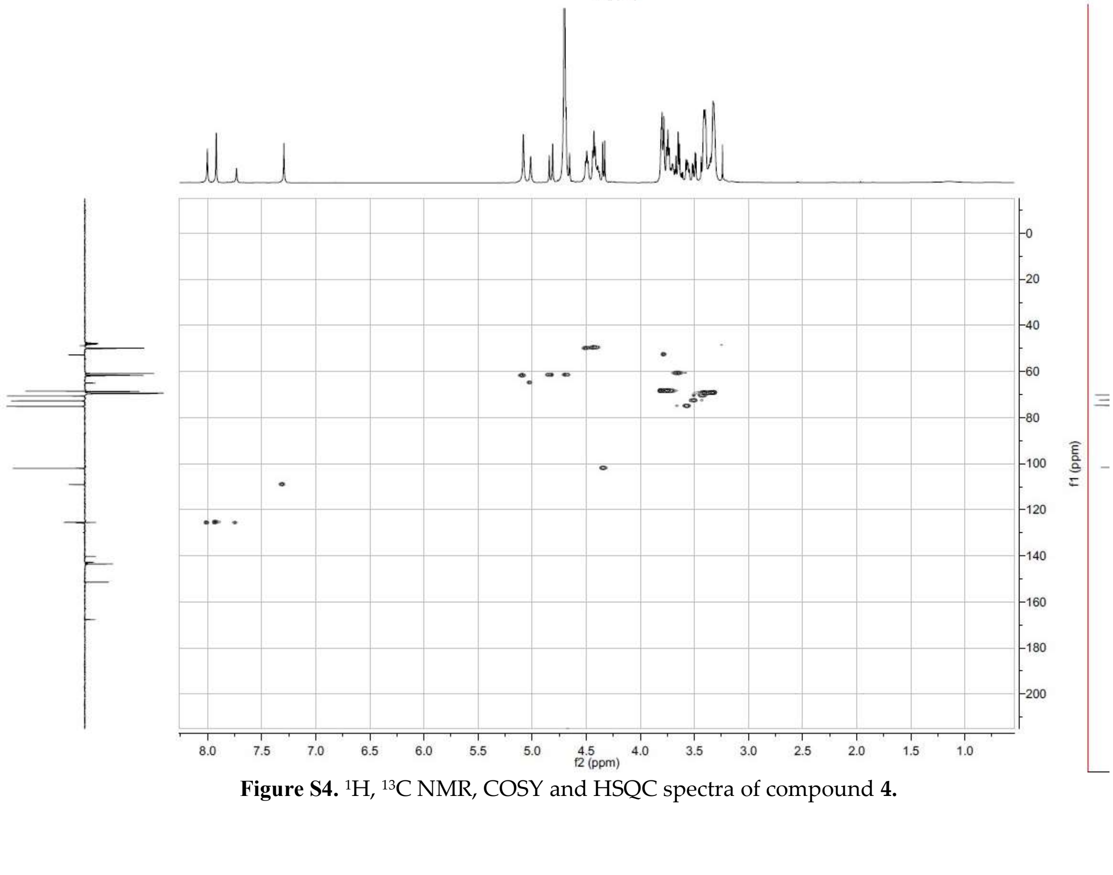

Figure S4. ^{1}H, ^{13}C NMR, COSY and HSQC spectra of compound **4.**

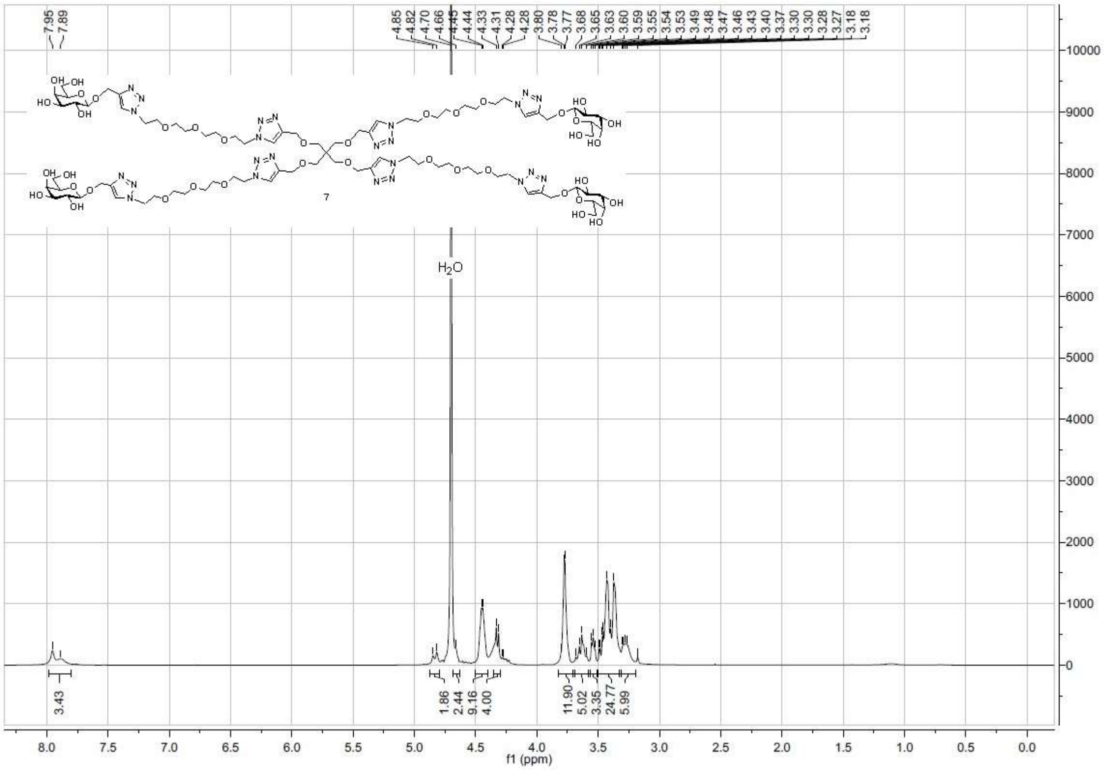
7.95
7.89
4.85
4.82
4.70
4.66
4.45
4.44
4.33
4.31
4.28
4.28
3.80
3.78
3.77
3.68
3.65
3.63
3.60
3.59
3.55
3.54
3.53
3.49
3.48
3.47
3.46
3.43
3.40
3.37
3.30
3.30
3.28
3.27
3.18
3.18
7
H2O
3.43
1.86
2.44
9.16
4.00
11.90
5.02
3.35
24.77
5.99
f1 (ppm)

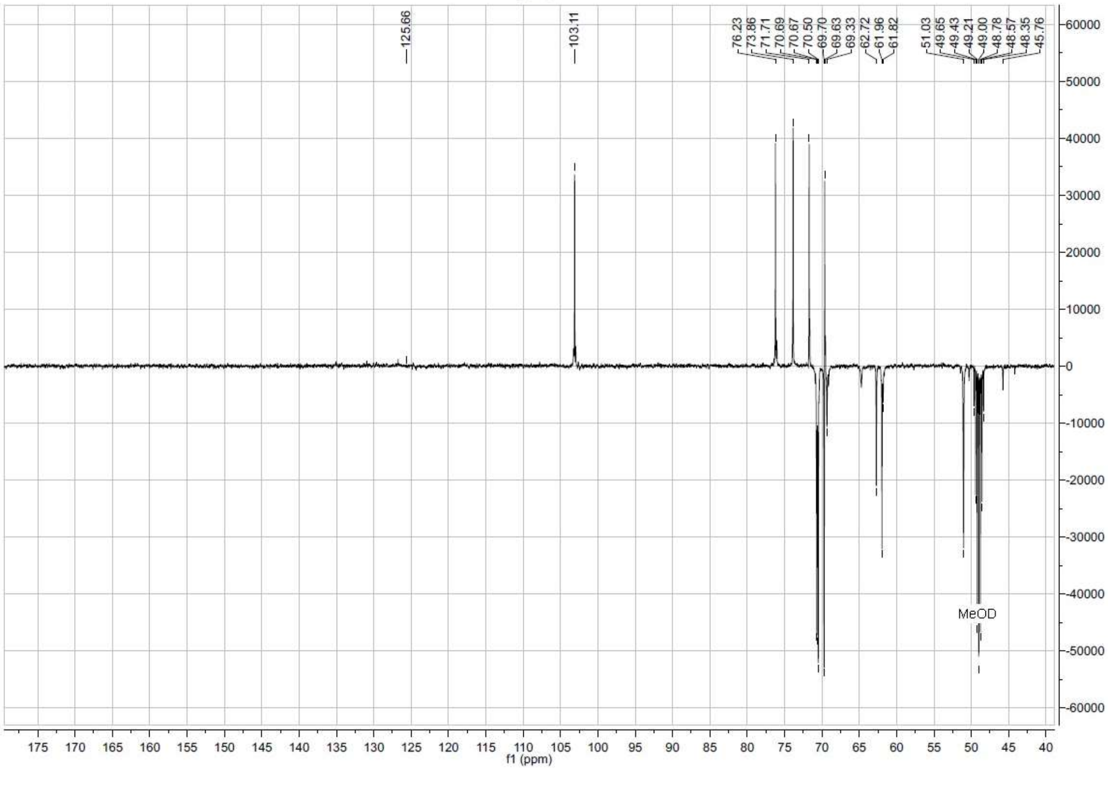
125.66
103.11
76.23
73.86
71.71
70.69
70.67
70.50
69.70
69.63
69.33
62.72
61.96
61.82
51.03
49.65
49.43
49.21
49.00
48.78
48.57
48.35
45.76
MeOD
f1 (ppm)

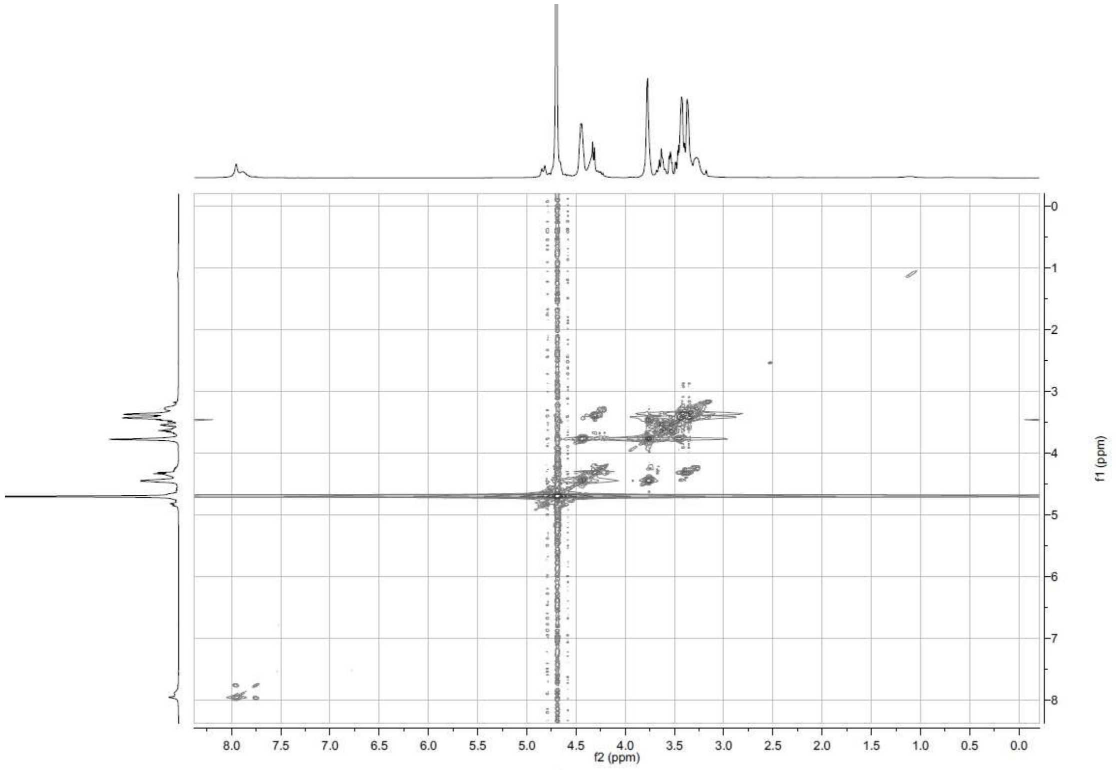

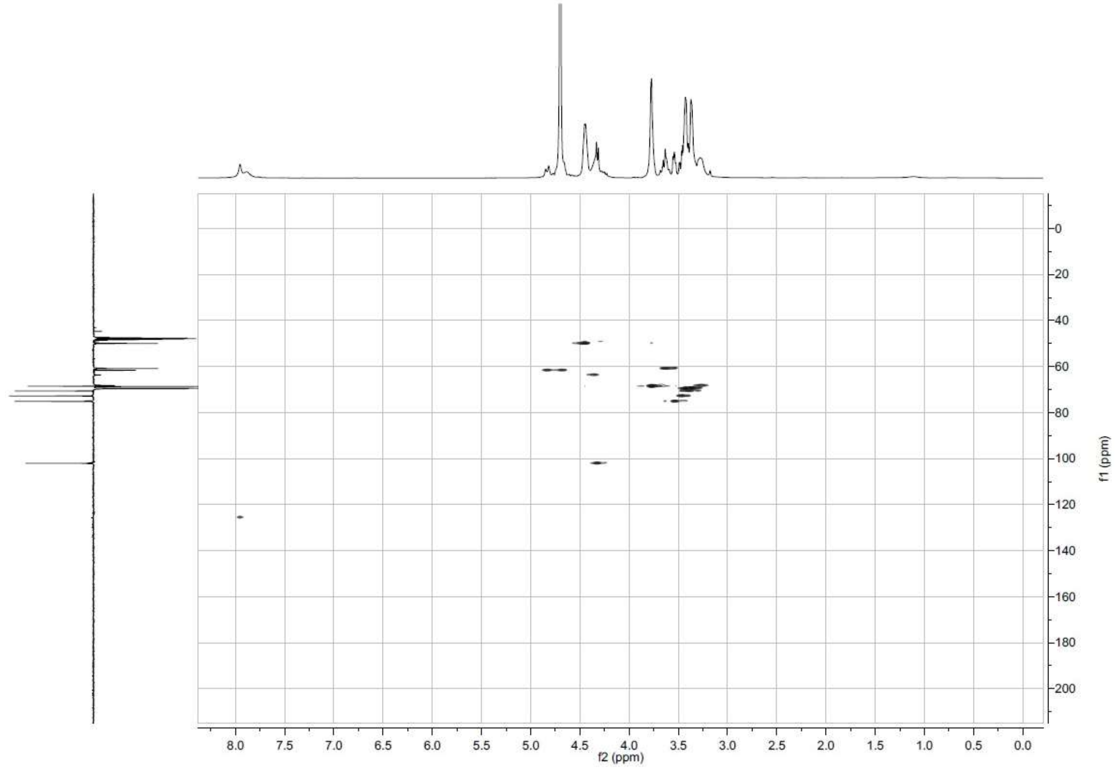

Figure S5. ^{1}H, ^{13}C NMR, COSY and HSQC spectra of compound **7.**

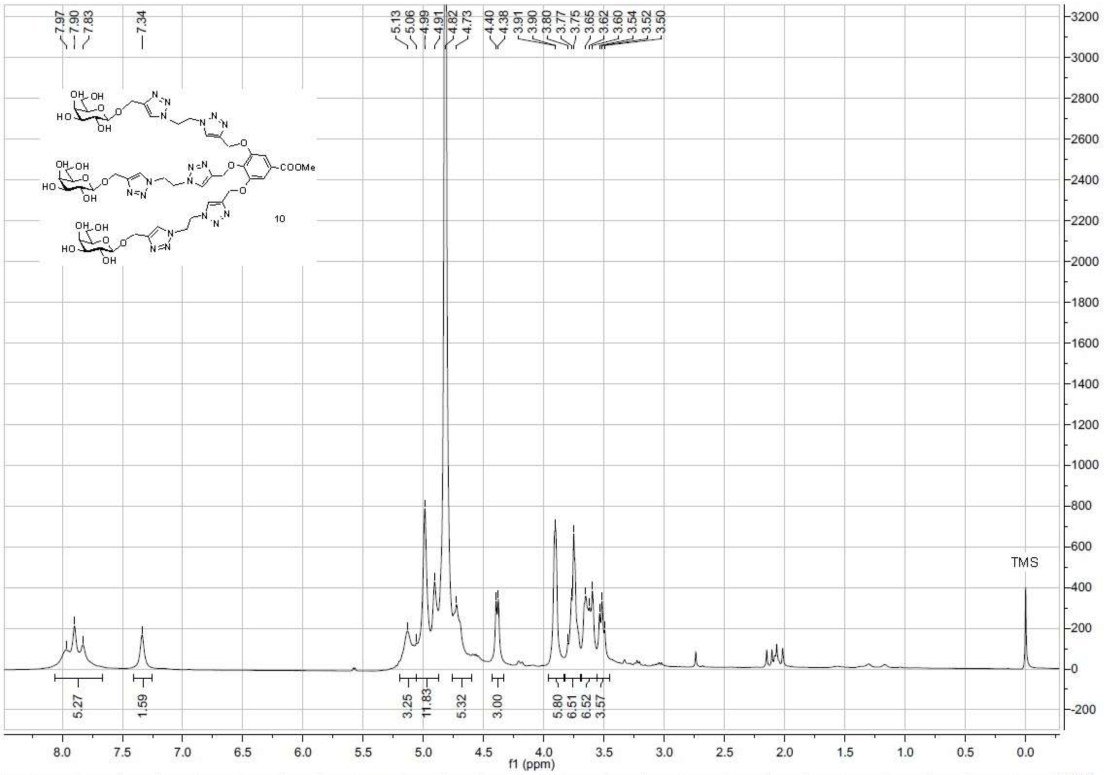
7.97
7.90
7.83
7.34
5.13
5.06
4.99
4.91
4.82
4.73
4.40
4.38
3.91
3.90
3.80
3.77
3.75
3.65
3.62
3.60
3.54
3.52
3.50
COOMe
10
TMS
5.27
1.59
3.25
11.83
5.32
3.00
5.80
6.51
6.52
3.57
f1 (ppm)

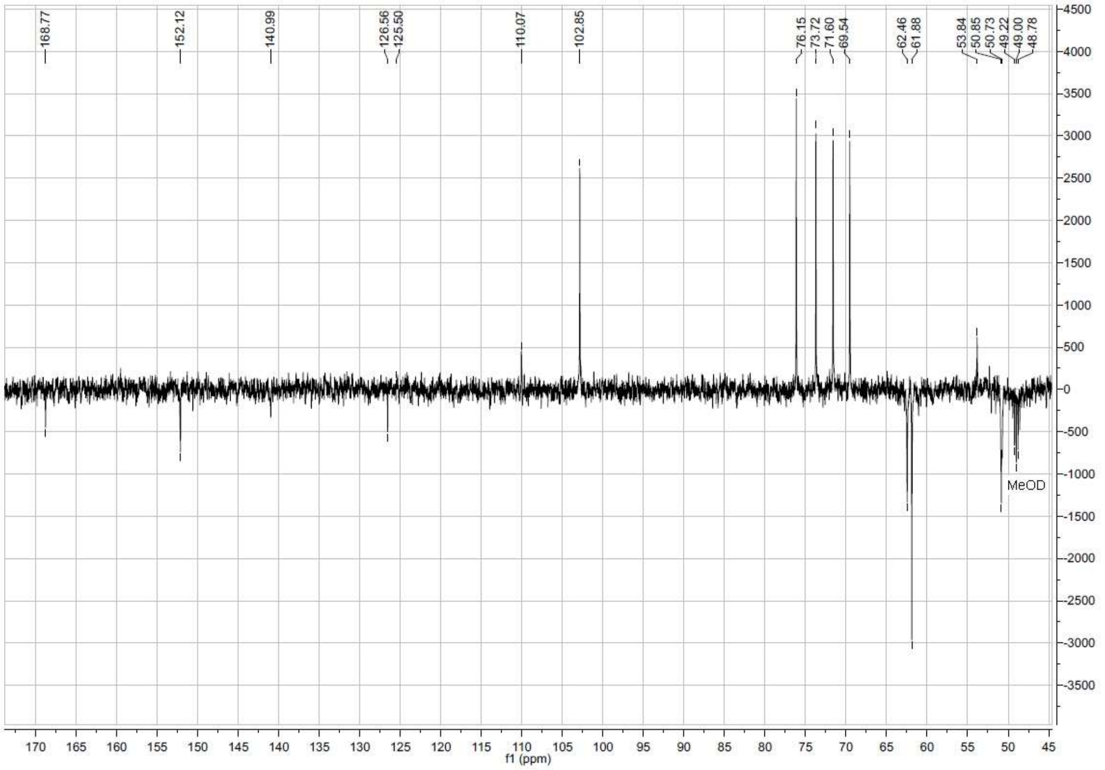
168.77
152.12
140.99
126.56
125.50
110.07
102.85
76.15
73.72
71.60
69.54
62.46
61.88
53.84
50.85
50.73
49.22
49.00
48.78
MeOD
f1 (ppm)

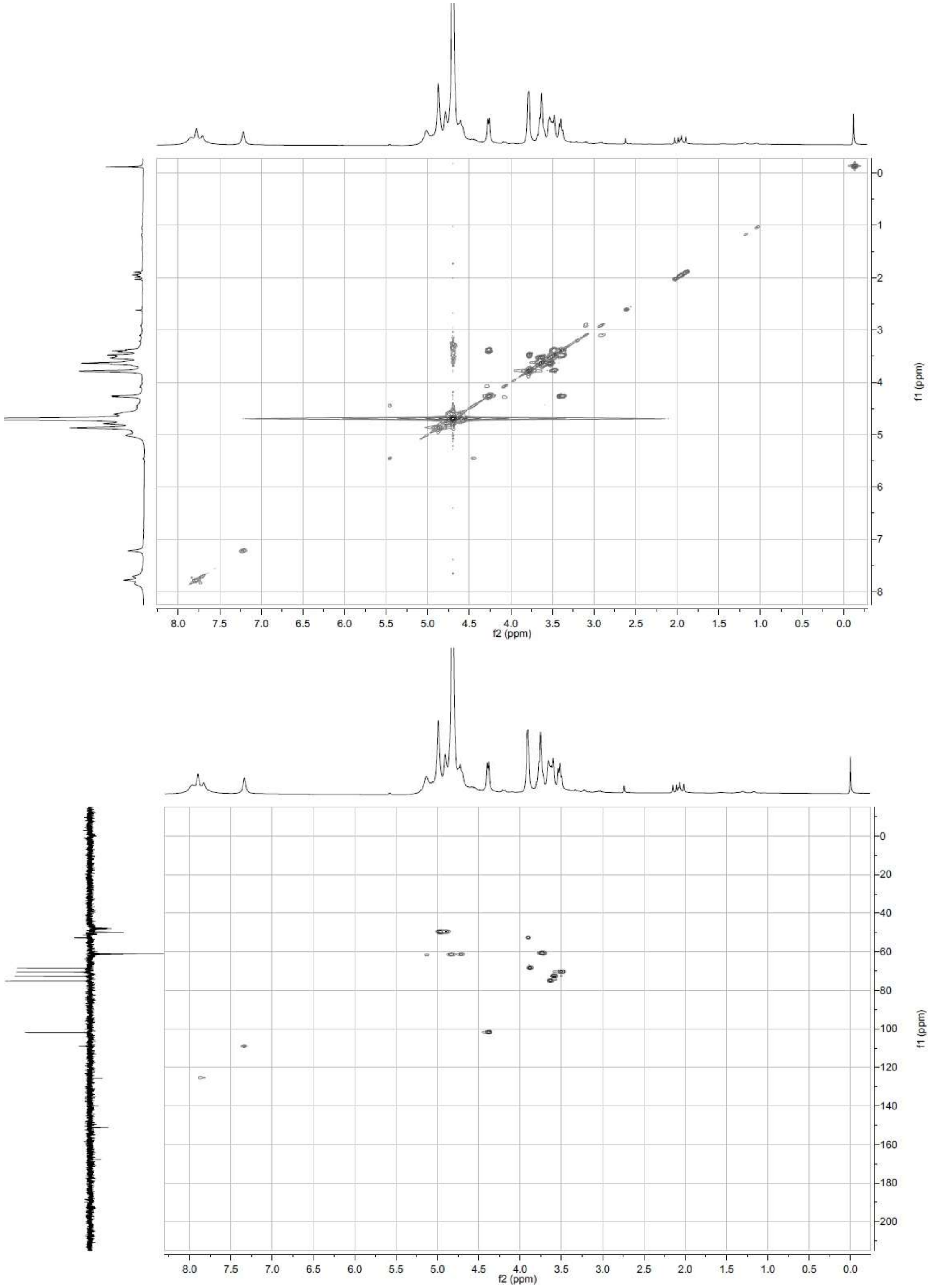

Figure S6. ^{1}H, ^{13}C NMR, COSY and HSQC spectra of compound **10.**

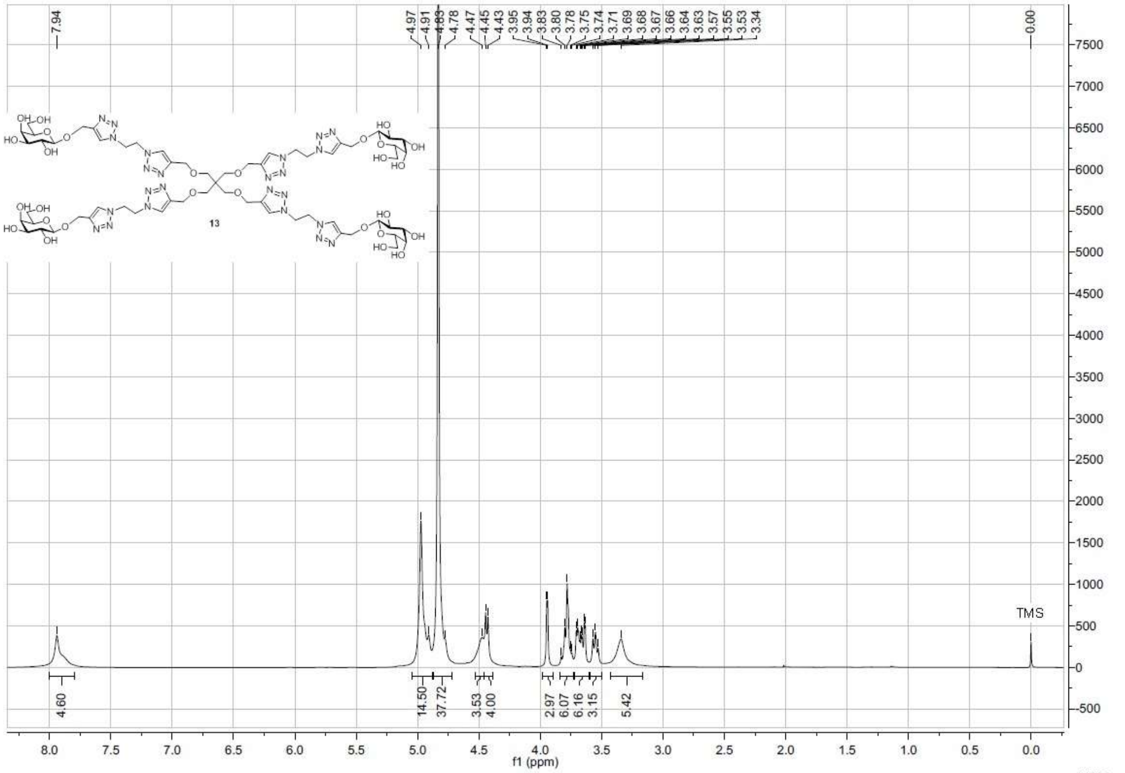
7.94
4.97 4.91 4.83 4.78 4.47 4.45 4.43 3.95 3.94 3.83 3.80 3.78 3.75 3.74 3.71 3.69 3.68 3.67 3.66 3.64 3.63 3.57 3.55 3.53 3.34
0.00
TMS
13
4.60
14.50 37.72 3.53 4.00 2.97 6.07 6.16 3.15 5.42
f1 (ppm)

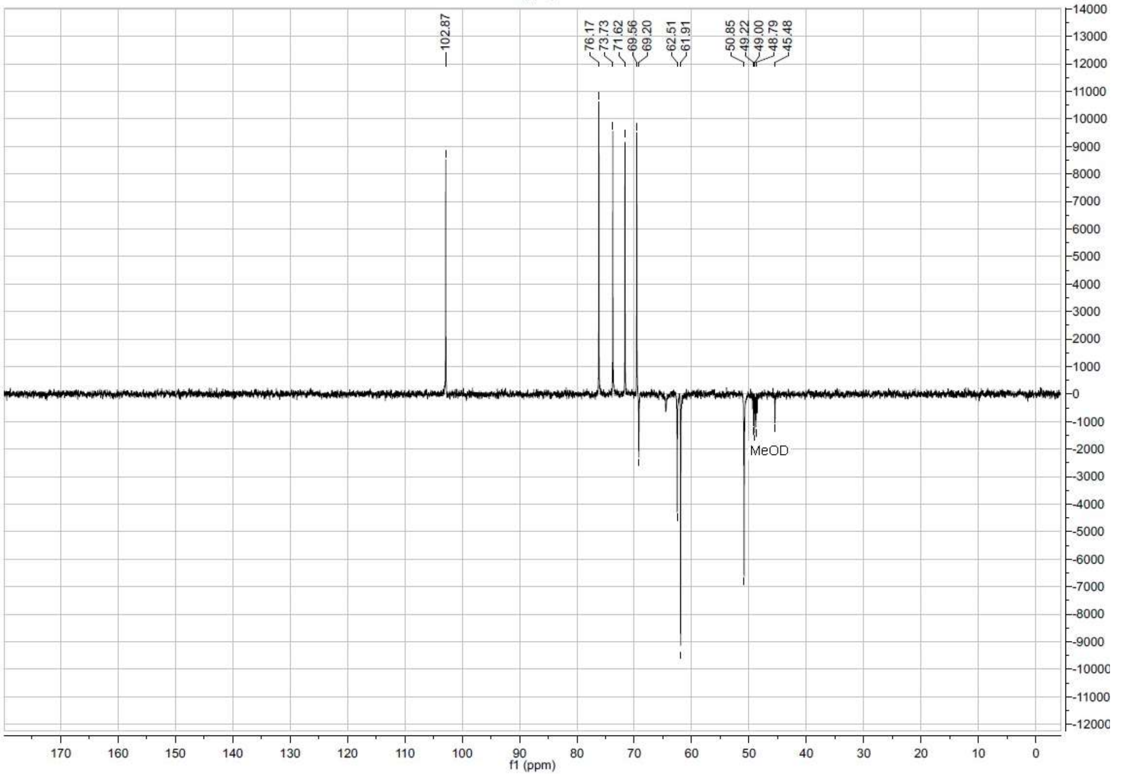
102.87
76.17 73.73 71.62 69.56 69.20 62.51 61.91
50.85 49.22 49.00 48.79 45.48
MeOD
f1 (ppm)

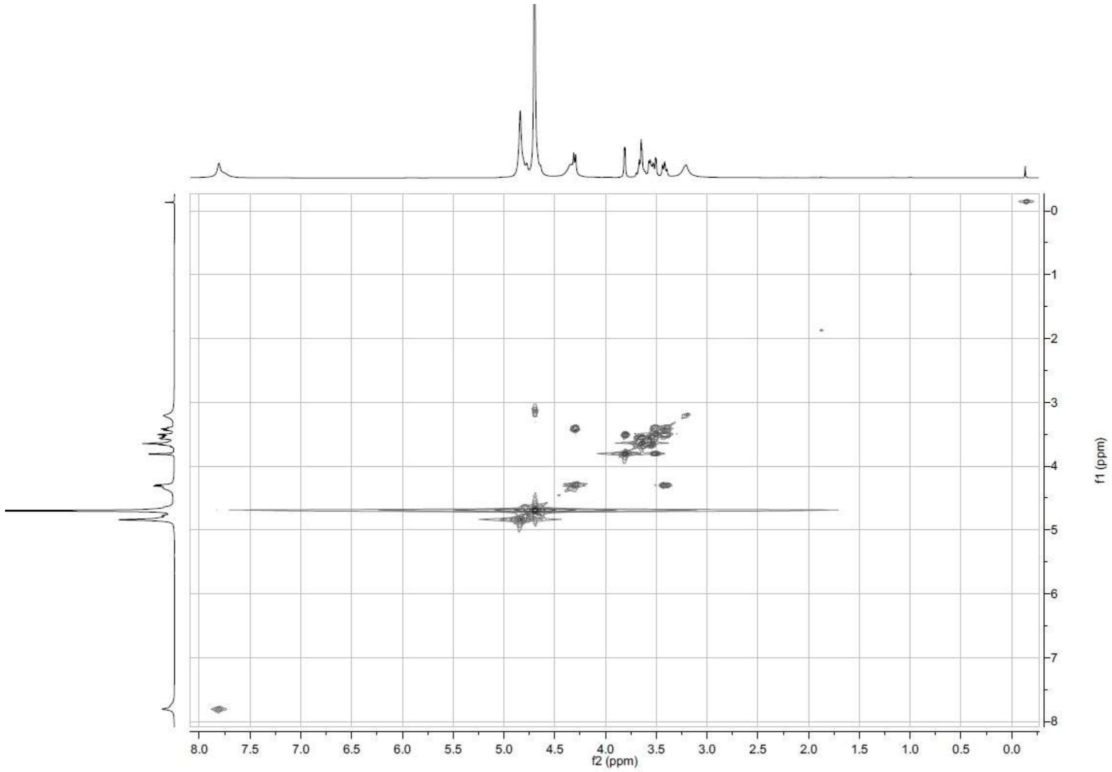

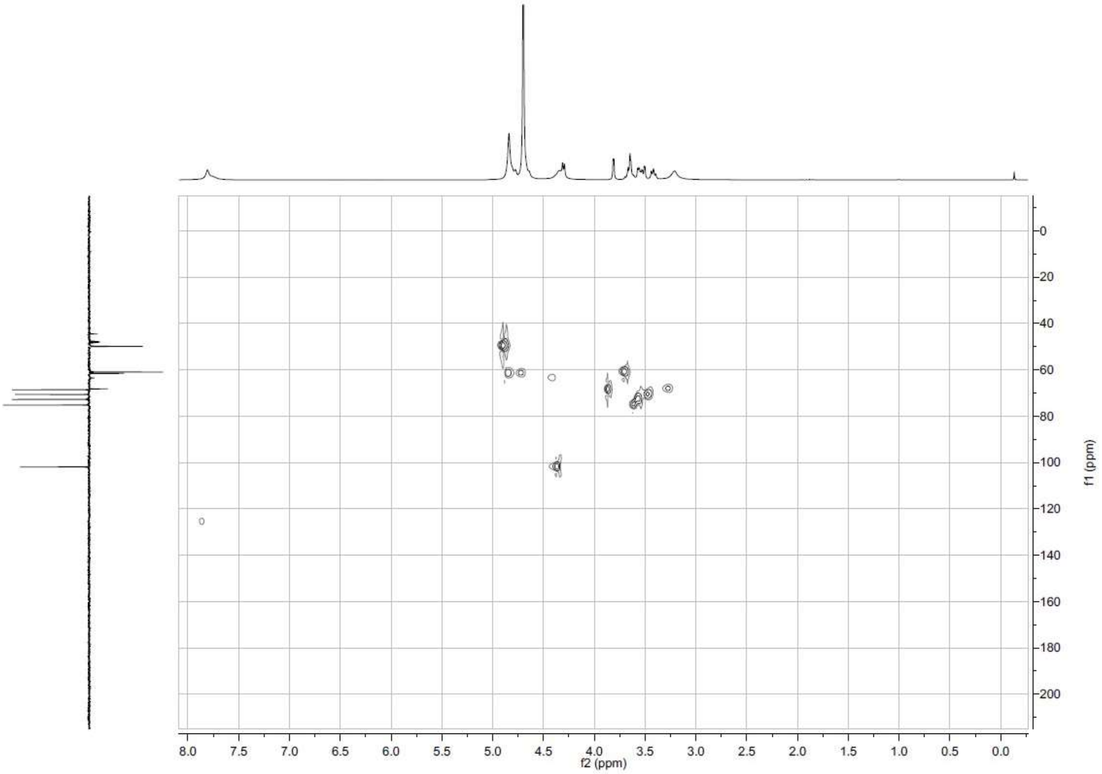

Figure S7. ^{1}H, ^{13}C NMR, COSY and HSQC spectra of compound **13.**
